# Design and synthesis of diazine-based panobinostat analogues for HDAC8 inhibition

**DOI:** 10.3762/bjoc.16.59

**Published:** 2020-04-07

**Authors:** Sivaraman Balasubramaniam, Sajith Vijayan, Liam V Goldman, Xavier A May, Kyra Dodson, Sweta Adhikari, Fatima Rivas, Davita L Watkins, Shana V Stoddard

**Affiliations:** 1 Department of Chemistry and Biochemistry, University of Mississippi, University, MS 38677-1848, USA; 2 Department of Chemistry, Rhodes College, Memphis, TN 38112, USA; 3 Department of Chemical Biology and Therapeutics, St. Jude Children’s Research Hospital, Memphis, TN 38105-3678, USA

**Keywords:** diazine, histone deacetylase, inhibitors, isozymes, panobinostat

## Abstract

Guided by computational analysis, herein we report the design, synthesis and evaluation of four novel diazine-based histone deacetylase inhibitors (HDACis). The targets of interest (TOI) are analogues of panobinostat, one of the most potent and versatile HDACi reported. By simply replacing the phenyl core of panobinostat with that of a diazine derivative, docking studies against HDAC2 and HDAC8 revealed that the four analogues exhibit inhibition activities comparable to that of panobinostat. Multistep syntheses afforded the visualized targets **TOI1**, **TOI2**, **TOI3-rev** and **TOI4** whose biological evaluation confirmed the strength of HDAC8 inhibition with **TOI4** displaying the greatest efficacy at varying concentrations. The results of this study lay the foundation for future design strategies toward more potent HDACis for HDAC8 isozymes and further therapeutic applications for neuroblastoma.

## Introduction

One of the most important posttranslational modifications involve acetylation/deacetylation of histone proteins by histone deacetylases (HDACs) [1]. HDACs belong to an important family of enzymes consisting of 18 isozymes. They control protein acetylation, which is a change that occurs after translation. In addition, they regulate gene transcription, cell differentiation, cell cycle progression and apoptosis by targeting both histone and non-histone proteins. The balance between acetylation and deacetylation is pivotal for typical cell function. Abnormal or increased HDAC expression has been reported in several human tumors and cancer cell lines [2]. As such, the development of novel HDAC inhibitors (HDACis) has become a rapidly evolving area where targeted inhibition has emerged in clinical research as a potential therapeutic approach for the treatment of various cancers as well as neurodegenerative disorders and immune related diseases [3–5]. Of specific interests are Class I HDAC isozymes, HDAC2 and HDAC8, which are important targets in cancer models as both are associated with high risk diseases such as prostate cancer and neuroblastoma [6–8]. Compounds such as vorinostat, givinostat and panobinostat have been successfully applied as HDAC inhibitors [3]. Among these drugs, panobinostat (Farydak, Novartis) an FDA approved drug, has been recognized as a pan-deacetylase inhibitor [9–10]. As a hydroxamic acid pan-HDACi, it is zinc-dependent, capable of binding in a bidentate fashion to the zinc-containing catalytic domain of the HDACs, and classified as highly potent amongst traditional HDACis [11]. According to previous reports, panobinostat not only induces apoptosis in cells, but also stimulates cell growth inhibition, and cell-cycle arrest in a time- and dose-dependent manner. Thus, panobinostat has demonstrated high therapeutic potential in anticancer efforts. Although panobinostat offers a versatile approach for the inhibition of cancer cell growth and survival, a lack of selectivity and bioavailability can cause negative molecular and clinical effects, specifically in combination therapies.

**Figure 1:**
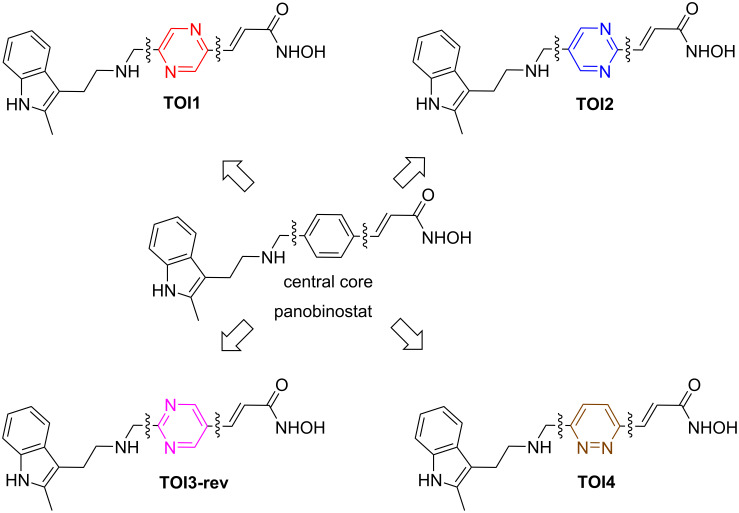
Chemical structures of the target diazine-based surrogates for the central core of panobinostat.

Despite advances in Class I HDAC inhibition, there remains an obvious need to develop compounds having better therapeutic properties as a single-agent therapeutic drug. Our recent research based on computational studies indicated heterocyclic cores as suitable surrogates for the central core of the hydroxamate derivative, panobinostat [12]. It should be noted that **TOI3-rev** in this article is different from **TOI3** in the previous reporting [12]. Here, the 1,2-diazole ring has been replaced with that of a pyrimidine core. Given the abundance of literature regarding analogues having modifications of the indole amine unit and vinylogous hydroxamic moieties [13–15], the non-availability of the central core modification stimulated our interest toward altering the central core to evaluate efficacy. We found particular interest in the replacement of the phenyl ring with diazine cores (pyridazines, pyrimidines and pyrazines) as their docking values were on par with that of the parent molecule, panobinostat (Figure 1). Given that the diazine-containing compounds are considered to be one of the most important classes of heterocycles, their presence in a plethora of pharmacology and drug molecules motivated us to synthesize these analogues and subject them towards biological evaluation [16–19]. In addition, our envisioned dinitrogen heterocycle cores may have increased interactions in the binding pockets, and thus leading to a better therapeutic activity.

Aiming to provide a strategy to address our global objective of developing single agent therapeutic hydroxamate derivatives, we began the synthesis of four leading HDAC8 diazine-based HDACis: **TOI1**, **TOI2**, **TOI3-rev** and **TOI4** (Figure 1). Herein, we provide a summary of the design process followed by an outline of the multistep synthesis and preliminary biological evaluation of each target. HDAC8 was selected for testing due to its unique structure and multifaceted functional activities [4–5,20–21]. HDAC8 is also upregulated in neuroblastoma, a childhood pediatric cancer, hence considered a drug target for this cancer subtype [22–23]. Despite a multifaceted array of alternative treatment options for neuroblastoma, some patient cohorts who are considered high risk at the time of diagnosis face poor prognosis [24–25]. Recent studies indicated that HDAC8 inhibition induces differentiated phenotypes and reduces neuroblastoma growth in vitro and in vivo with few adverse effects [22]. However, there are very few effective therapeutic options in neuroblastoma that inhibit HDAC8 [26]. Thus, the design of novel HDAC8 inhibitors as potential neuroblastoma therapeutics could be valuable to expand the treatment options for this patient population [27]. Therefore, we sought to focus on the biological evaluation of our proposed inhibitors in HDAC8. This study aimed at offering additional therapeutic options to be used in conjunction with or in place of panobinostat while providing a rationale design guideline towards HDACis.

## Results and Discussion

### Molecular design

Docking analysis is a well-established technique that is utilized to evaluate interactions in important biological receptors such as that of HDACs [28–30]. Previously, we implored docking to predict the interactions between the active sites of HDAC2 and HDAC8 molecular frameworks with similar structures to that of panobinostat [12]. In developing such analogues, the hydroxamate tails and indole capping moiety were maintained as both are essential to binding at the active site of HDAC2 and HDAC8. Results suggested that **TOI1**, **TOI2**, and **TOI4** [12] would be inhibitors exhibiting similar potency as that of panobinostat. The reported −log(*K*_d_) values were 8.93, 8.64 and 8.25, respectively, with panobinostat possessing a docking score of 8.47. Considering the effects of structural diversity, **TOI3-rev** was included in the library and computationally evaluated to determine its potential as an HDAC8 inhibitor. **TOI3-rev** possesses a −log(*K*_d_) value of 8.36 suggesting that it too would be on a par with panobinostat (8.47).

**Figure 2:**
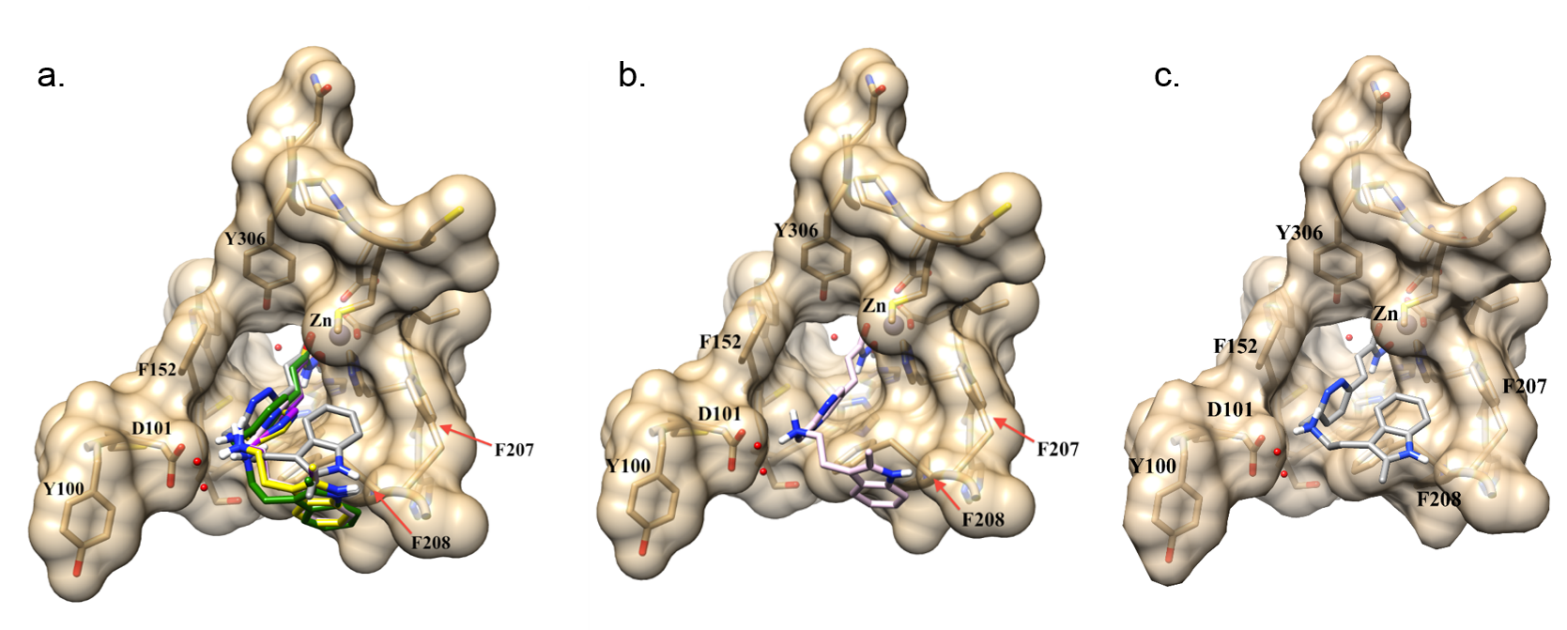
Docking pose for panobinostat and panobinostat derivatives in the HDAC8 receptor. (a) Overlay of all compounds investigated in this study in the HDAC8 active site: panobinostat (green), **TOI1** (purple), **TOI2** (yellow) and **TOI4** (grey); (b) **TOI3-rev** (pink) docking pose in active site; (c) **TOI4** (grey) docking pose in the active site.

Our previous study showed that the hydroxamate tail of **TOI1**, **TOI2**, and **TOI4** formed a bidentate interaction with the Zn^2+^ ion at the base of the HDAC8 receptor, which is consistent with the crystal structure of this class of inhibitors in HDACs [31–34]. Again, our work here extends the investigation focusing on HDAC8 due to the need for novel neuroblastoma therapeutics. Each compound was also shown to have two parallel-displaced π–π interactions, one with Phe-152 and the other with Phe-208. The mode of binding for each compound was similar to panobinostat in HDAC8 (Figure 2). Two differences were however observed in the mode of binding of **TOI4**. It was shown that the pyridazine ring did not lay as planar in the gorge as the pyrazine and pyrimidine rings of **TOI1** and **TOI2**, respectively. The indole ring of **TOI4** was also contorted upward, forming a T-shaped π–π interaction with Phe-207 while both the indole rings of **TOI1** and **TOI2** were shown to be flipped downward forming a T-shaped π–π interaction with Phe-208.

**TOI4** is the only diazine compound having two nitrogens directly next to each other in the core ring structure. The nitrogen atoms in the pyridazine ring are positioned so that they do not sit in the center of the phenyl rings of Phe-152 or Phe-208. This results in the pyridazine ring of **TOI4** shifted closer to the side of the gorge having Phe-152 compared to the other derivative (Figure 2). This shift allows for the indole ring of **TOI4** to fit in a small hydrophobic pocket at the surface, which is created by the phenyl ring of Phe-207. Like the previously studied compounds (**TOI1**, **TOI2**, and **TOI4**), **TOI3-rev** is also shown to bind to the HDAC8 receptor by forming a bidentate interaction with the Zn^2+^ ion. **TOI3-rev**, like that of **TOI1**, **TOI2**, and **TOI4** produces two parallel-displaced π–π interactions with Phe-152 and Phe-208 (Figure 2). The pyrimidine ring of **TOI3-rev** also lays planar in the gorge similar to the pyrazine and pyrimidine rings of **TOI1** and **TOI2**. The indole ring of **TOI3-rev** is tilted downward forming a T-shaped π–π interaction with Phe-208.

In review of the computational results, the inclusion of the nitrogen atoms in the core ring structure of panobinostat produced compounds with predicted binding affinities similar to panobinostat. Thus, aiming to develop improved compounds to effectively target HDAC8, the synthesis of TOI inhibitors and their evaluation was undertaken.

### Synthesis

**Figure 3:**
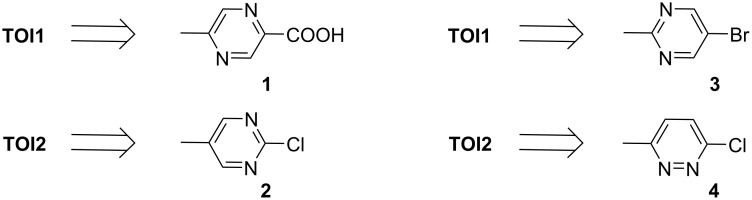
General building blocks for the visualized targets.

Equipped with molecular targets achieved via theory, the synthesis of the hypothesized compounds commenced with commercially available starting materials (Figure 3). The central core building blocks for **TOI1**, **TOI2**, **TOI3-rev** and **TOI4** were selected based on their ability to tether the indole amine and the zinc binding group in a *para*-relationship to each other.

**Scheme 1:**
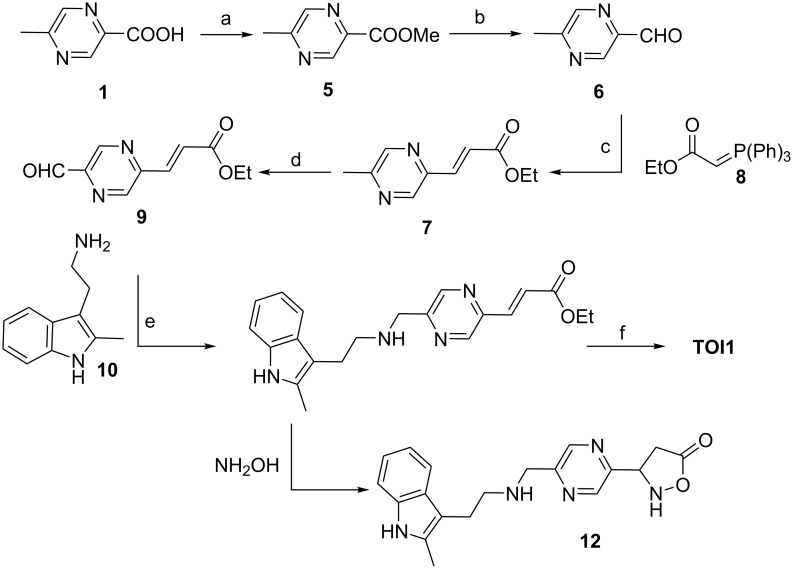
Reaction conditions: a) MeOH, H_2_SO_4_ (5 drops), MS 4 Å (2 pieces), 68 °C, 8 h, 81%; b) DIBAL-H (1.2 equiv), 6 h, −78 °C, THF, 78%; c) phosphorane **8** (2.0 equiv), THF, 8 h, 60 °C, 72%; d) SeO_2_, dioxane, 110 °C, 8 h, 61%; e) indolamine **10** (1.1 equiv) DCE, sodium triacetoxyborohydride (STAB, 1.1 equiv), TEA (2 equiv), rt, 63%; f) NaOH at −10 °C, NH_2_OH·H_2_O at −10 °C, MeOH , rt, 12 h, 55%.

Initial efforts focused on synthesizing analogue **TOI1** (Scheme 1). Acid **1** was converted to the corresponding methyl ester **5** via esterification reaction using methanol mediated by sulfuric acid under heating conditions to provide the compound in 81% yield. The methyl ester **5** was reduced to aldehyde **6** using DIBAL-H at −78 °C. While TLC analysis revealed complete conversion of the ester to aldehyde, the isolated yield was poor (20%). The obstacle was overcome by using a modified Fieser work-up procedure to yield the aldehyde **6** in high yield, 78%. Then, aldehyde **6** was converted to the α,β-unsaturated *trans*-ester **7** through a Wittig reaction with the phosphorane synthon **8**, which was derived from ethyl bromoacetate at 60 °C for 8 h in THF in 72% yield. The exclusive formation of the *trans-*isomer was confirmed by ^1^H NMR studies, namely the presence of the olefin at δ 7.00 and 7.71 with a *J* value of 15 Hz (Supporting Information File 1, Figure S3).

Next, oxidation of the methyl group of **7** under SeO_2_ conditions at 110 °C provided the ethyl acrylate aldehyde **9** in 61% yield. The next step involved the crucial reductive amination reaction between aldehyde **9** with indolamine **10**, which had been obtained via Fischer indole synthesis – the reaction of phenylhydrazine with 5-chloro-2-pentanone [35]. Initial reduction attempts using sodium triacetoxyborohydride (STAB) as the reducing agent provided predominantly starting material and negligible potential product as monitored by TLC. However, addition of 2 equivalents of triethylamine to the reaction mixture facilitated the formation of the product, compound **11** in 63% yield. The product was confirmed by ^1^H NMR and ^13^C NMR as shown in Supporting Information File 1.

Finally, the ethyl ester **11** was converted to the hydroxamic acid derivative, **TOI1** using the bidentate nucleophile hydroxylamine either under neutral or basic conditions [36–37]. We first explored neutral conditions where aqueous hydroxylamine was added to compound **11** in methanol, and a predominant polar spot was observed by TLC. However, the isolated product was not the expected **TOI1**, as ^1^H NMR revealed two new peaks at δ 4.53 and 2.66 ppm (Supporting Information File 1, Figure S36) presumably indicating that a favorable Michael addition followed by intramolecular cyclization or vice versa provided compound **12**, which was validated by ^13^C and DEPT NMR studies (Supporting Information File 1 Figures S37and S38).

Marred with these observations, compound **11** was treated with aqueous hydroxylamine in the presence of strong base (i.e., 10 equivalents of methanolic sodium hydroxide or aqueous sodium hydroxide) at 0 °C. The reaction was monitored by TLC and it revealed that methanolic sodium hydroxide provided a cleaner reaction than aqueous sodium hydroxide conditions. The reaction mixture was quenched with a saturated ammonium chloride solution at 0 °C after 12 h, the solvent was evaporated, and the compound was subjected to reversed-phase column chromatography using C-18 silica gel. After initial unsuccessful purification protocols with water/ACN or water/THF solvent systems, we identified an optimized water/methanol mixture to provide the pure product **TOI1** in 55% yield. The isolated compound was thoroughly characterized by spectroscopic techniques.

**Scheme 2:**
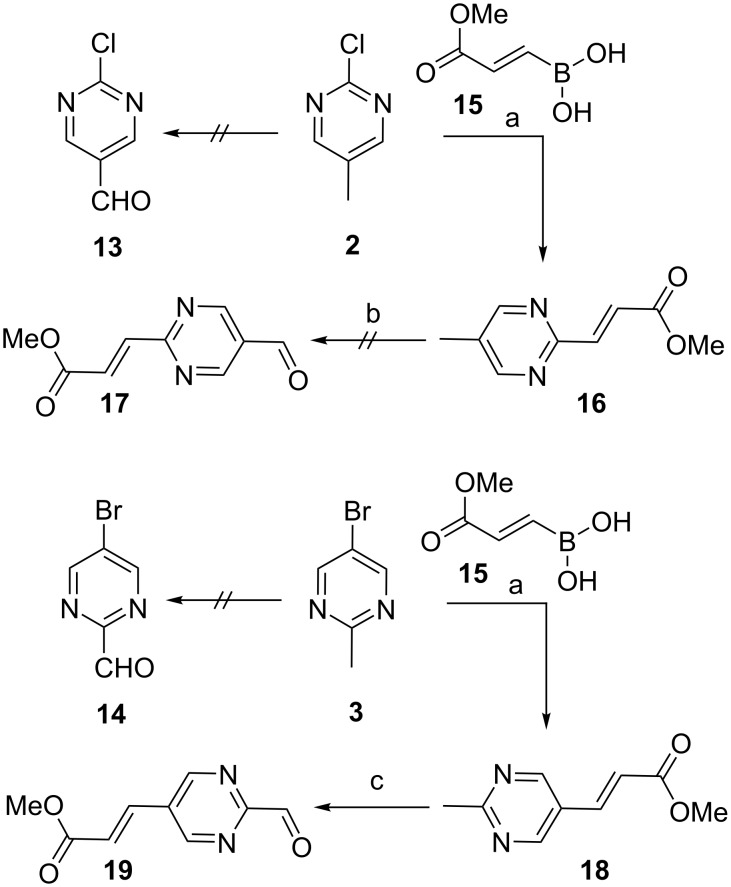
Reaction conditions: a) boronic acid **15** (1.3 equiv), PdCl_2_(PPh_3_)_2_ (0.1 equiv), dioxane/H_2_O (3:1), Na_2_HPO_4_ (2.0 equiv), TEA (4.0 equiv), 90 °C, 15 h, 55% for **16** and 71% for **18**; b) SeO_2_ , different conditions, 0%; c) SeO_2_ (2.0 equiv), dioxane, 110 °C, 12 h, 54%.

Having successfully establish reaction conditions for the synthesis of **TOI1**, we then focused our efforts on the generation of regioisomers **TOI2** and **TOI3-rev**, respectively. Initial attempts to oxidize the methyl group at the benzylic position in starting materials **2** and **3** to provide the corresponding aldehyde compounds **13** and **14** failed, despite using rigorous reaction conditions of SeO_2_ or alternative strong oxidizing agents (e.g., MnO_2_ and oxone). Thus, we considered the critical role of the electronic effects of the nitrogen atoms on this cyclic substrate, and then we revised our synthetic strategy by a) tethering an alkene functional group on the aromatic ring and b) then conducting the oxidation of the benzylic group to afford the aldehyde product. Towards this end, we performed a Suzuki coupling reaction between boronic acid **15** with chloro compound **2** (Scheme 2). To the best of our knowledge, there is no report of a Suzuki coupling reaction using boronic acid **15** in the literature. However, we generated this required boronic acid from the corresponding methyl propiolate [38].

Next we investigated reaction conditions for the reaction of compound **2** with boronic acid **15** using different variables (Supporting Information File 1, Table S1). Gratifyingly, after surveying several reaction conditions, we successfully obtained the desired product **16** in 35% yield using PdCl_2_(PPh_3_)_2_ and Na_2_HPO_4_ as the base in a dioxane/water system under heating conditions of 90 °C.

To improve the overall chemical yields, we evaluated several organic bases and found that TEA (4.0 equiv) improved the yield to 55%. This success was attributed to the improved increased solubility of TEA in the reaction mixture under heating conditions. In parallel, similar reaction conditions were used for compound **3** and the final coupled product **18** was obtained in 71% yield. The product was confirmed by the expected chemical shift at δ 6.50 and 7.54 ppm with a *J* value of 15 Hz for compound **16** (Supporting Information File 1, Figure S7) and δ 6.57 and 7.63 ppm with a *J* value of 15 Hz for compound **18** (Supporting Information File 1, Figure S9) as inferred by ^1^H NMR analysis.

The resulting Suzuki-coupled products **16** and **18**, were subjected to benzylic oxidation expecting the olefin functionality would facilitate the corresponding aldehydes **17** and **19**, respectively. Surprisingly, the methyl group in compound **16** did not undergo oxidation under SeO_2_ conditions as observed for **TOI1** whereas under the same reaction conditions, compound **18** readily underwent oxidation to yield compound **19** in 54% yields.

**Scheme 3:**
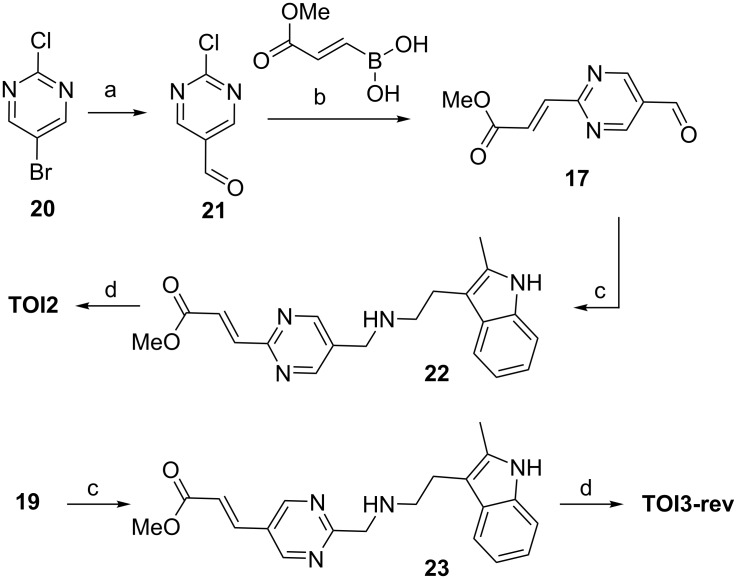
Reaction conditions: a) 5-bromo-2-chloropyrimidine (1 equiv), ethyl formate (1.5 equiv), THF (20 mL), *n*-butyllithium (0.6 equiv, 2.5 M) in hexane, −100 °C, 2 h, 42%; b) boronic acid (1.3 equiv), PdCl_2_(PPh_3_)_2_ (0.1 equiv), dioxane/water (8:2), Na_2_HPO_4_ (2.0 equiv), TEA (4.0 equiv), 95 °C, 15 h, 40%; c) indolamine **10** (1.1 equiv) DCE, STAB (1.0 equiv), TEA (2 equiv), rt, 61% for **22**, 68% for **23**; d) NaOH at −10 °C, NH_2_OH·H_2_O at −10 °C, MeOH, rt, 12 h, 49% for **TIO2** and 51% for **TIO3-rev**.

Confounded by this observation, extensive experimentation varying solvent and temperature were evaluated, but none were fruitful. Alternatively to synthesize compound **17**, we adapted the strategy as depicted in Scheme 3. Compound **20** was converted into compound **17** via intermediate **21** using a previously reported literature procedure [39]. The intermediate **21** was then subjected to the Suzuki reaction using conditions already developed to provide the ester aldehyde **17** in overall yield of 16% for two steps. Having identified synthetically suitable conditions for compound **17**, we scaled up the reaction to complete the final two steps, the reductive amination reaction and the hydroxamic acid preparation. Using the same reaction conditions developed for **TOI1**, we proceeded with precursors **22** and **23**, which were obtained in 61% and 68% yield, respectively. The desired hydroxamic acid **TOI2** and **TOI3-rev** were obtained in 49% and 51% yield, respectively, after C18 silica gel purification procedure using a methanol/water (1:1) solvent system.

**Scheme 4:**
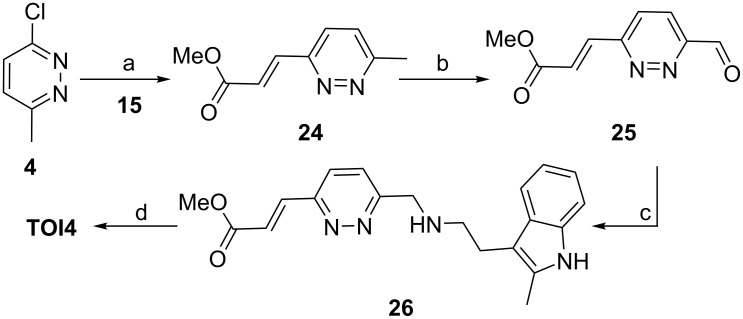
Reaction conditions: a) boronic acid **15** (1.3 equiv), PdCl_2_(PPh_3_)_2_ (0.1 equiv), dioxane/H_2_O (8:2, Na_2_HPO_4_ (2.0 equiv), TEA (4.0 equiv), 95 °C, 15 h, 41%; b) SeO_2_ (2.0 equiv), dioxane , 110 °C, 16 h, 52%; c) indolamine **10** (1.1 equiv), DCE, STAB (1.0 equiv), TEA (2 equiv), rt, 38%; d) NaOH at −10 °C, NH_2_OH·H_2_O at −10 °C, MeOH, rt, 12 h, 44%.

Finally, we focused our efforts towards the synthesis of the last analogue **TOI4**. From the above observations, we hypothesize that successful synthesis of **TOI4** would rely on generating the key intermediate aldehyde **25** (Scheme 4).

Initially, we explored the Suzuki coupling reaction for substrate **4**. Gratifyingly, the reaction product **24** was produced in 41% yield from our developed synthetic strategy. Then, compound **24** was subjected to an SeO_2_ oxidation reaction. The oxidation reaction was performed at 110 °C for 16 h to furnish the aldehyde **25** in 52% yield. Next, successful coupling of the aldehyde **25** with indolamine **10** yielded the expected product **26** in 38% yield. Compound **26** was converted to hydroxamic acid **TIO4** in 44% yield under NaOH conditions in methanol and purified using C18 column chromatography. All the final compounds were thoroughly characterized by NMR and mass spectrometry (see Supporting Information File 1).

Having successfully synthesized the targets **TOI1** to **TOI4**, our next aim was to evaluate them biologically.

### Inhibition assay

The biochemical evaluation of the proposed inhibitors **TOI1**, **TOI2**, **TOI3-rev**, and **TOI4** was performed to experimentally determine the potency of HDAC8 inhibition. The obtained results, as shown in Table 1, aligned with the predicted computational studies. In a homogeneous fluorogenic assay, the HDAC activity is quenched with a fluorescent dye that is tethered to an acetyllysine-containing peptide. If the acetyl moiety of the fluorophore is enzymatically hydrolyzed by HDAC8, it will produce a strongly fluorescent signal at 360 nm. Table 1 shows the percentage of HDAC8 inhibition at 100, 10, and 1 µM concentration of the designed inhibitors and panobinostat. All compounds displayed HDAC8 inhibition as predicted by our computational studies [12]. Complete inhibition of HDAC8 was observed for panobinostat at 100 µM or 10 µM concentration, while only 89% inhibition was recorded at 1 µM concentration. The in vitro IC_50_ values for panobinostat against HDAC8 have been shown to be 277 nM [11]. In vivo studies measuring the IC_50_ values of panobinostat have also been performed, however, since panobinostat is a pan-DAC inhibitor it has been difficult for researchers to specifically correlate its IC_50_ value for HDAC8 in a physiological system [40]. Theoretical studies predicted **TOI1** as the most potent inhibitor over **TOI2**, **TOI3-rev**, and **TOI4**. **TOI1** was shown to be the most potent inhibitor designed in this study producing 100% inhibition at a 100 µM, 90% inhibition at a 10 µM, and 44% at a 1 µM concentration. At a 100 µM concentration, **TOI2**, **TOI3-rev** and **TOI4** inhibited 79%, 89%, and 93% of HDAC8 activity, respectively. Strong inhibition was seen at a 10 µM concentration for all inhibitors. At a concentration of 1 µM all inhibitors showed less inhibition of HDAC8 than panobinostat. It was noted that **TOI2** and **TOI3-rev** produced similar inhibition results against HDAC8. Both of these inhibitors have pyrimidine rings in the core; thus, the data suggests that while this ring structure did produce inhibition, it was not as effective as the inclusion of a pyrazine ring. This finding is consistent with our computational data which demonstrated that **TOI1** would slightly outperform **TOI2** and **TOI3-rev**.

**Table 1:** Percent inhibition of HDAC8 by hydroxamate inhibitors.

compound	concentration		

	100 µM	10 µM	1 µM

panobinostat (*n* = 4)^a^	100 ± 0.0	100 ± 0.0	89 ± 13.4
**TOI1** (*n* = 4)	100 ± 0.0	90 ± 21.1	44 ± 11.5
**TOI2** (*n* = 4)	79 ± 8.1	75 ± 13.4	23 ± 26.9
**TOI3-rev** (*n* = 4)	89 ± 7.0	75 ± 26.9	37 ± 18.1
**TOI4** (*n* = 4 @ 100 and 10 µM, *n* = 3 @ 1 µM)	93 ± 8.1	89 ± 13.4	65 ± 21.4
TCA^b^	93 ± 14.0		

^a^*n* = number of replicates for the assay; ^b^trichostatin A (TCA) as an inhibitor control.

It is interesting to note that **TOI1**, which was shown to be better than **TOI4** at the 100 µM level, was less potent than **TOI4** at low concentration (1 µM). **TOI4**, which was predicted to be slightly less potent than **TOI1**, **TOI2**, and **TO3-rev** computationally was revealed via experiment to be a better inhibitor than **TOI2** and **TOI3-rev**. The result was observed presumably due to a variation in the binding mode for **TOI4** that differs from that of **TOI1**, **TOI2**, and **TOI3-rev**. **TOI4** was shown to observe a slightly different orientation of the core heterocyclic ring as well as a change in the orientation of the terminal indole ring. It is not clear if this difference in binding mode affected the ability of **TOI4** to maintain a higher potency at lower concentrations. However, this observation does raise an interesting question: “Are certain modes of binding more effective at different concentrations and if so how can this be accurately modelled computationally?” Taken together, this data indicates that while **TOI1** is the best inhibitor of the four compounds presented herein, **TOI4** remains effective against HDAC8 even at lower concentrations.

## Conclusion

Our investigation provides a successful synthetic strategy towards four new analogues of panobinostat having diazines as the central core and details the results of their biochemical evaluation. Computational data corroborated that the substitution of benzene in the molecular framework of panobinostat for a nitrogen-containing heterocycle in the core ring structure would enhance the pharmacological properties while maintaining the level of HDAC8 inhibition. The targets **TOI1**–**4** were synthesized from commercially available starting materials in moderate yields. The synthesized compounds displayed potent activity against HDAC8; thus, emphasizing the advantages of drug design on a theoretical basis. These efforts led to the design of potential analogues that warrant further studies to develop therapeutic agents for neuroblastoma. Future research will be aimed at investigating the HDAC class specificity of these designed analogues and evaluating their overall potential to inhibit neuroblastoma cell growth.

## Experimental

### General

Reagents and solvents were purchased from commercial sources and used without further purification unless otherwise specified. Tetrahydrofuran (THF), ether, dichloromethane (DCM), and dimethylformamide (DMF) were degassed in 20 L drums and passed through two sequential purification columns (activated alumina; molecular sieves for DMF) under a positive argon atmosphere. Thin-layer chromatography (TLC) was performed on SiO_2_-60 F254 aluminum plates with visualization by ultraviolet (UV) detection at 254 nm or staining. Flash column chromatography was performed using Purasil SiO_2_-60, 230–400 mesh from Whatman. NMR spectra were recorded on a BRUKER AV500 spectrometer (operating at 500 MHz for ^1^H and 125 MHz for ^13^C acquisitions). Chemical shifts were reported as ppm relative to the solvent residual peak (CHCl_3_: 7.26 ppm for ^1^H, 77.2 ppm for ^13^C). Data are reported as follows: chemical shifts, multiplicity (s = singlet, d = doublet, t = triplet, q = quartet, quint = quintet, m = multiplet, br = broad), coupling constant *J* (Hz), and integration. High-resolution mass spectra were recorded using an ESI–TOF mass spectrometer (Agilent 6220 Time-of-Flight), gas temperature – 350 °C, drying gas (N_2_) – 8.0 L/min, mobile phase(s): methanol with 0.1% formic acid, flow rate: 0.2 mL/min, sample preparation: The sample was dissolved in 1 drop of chloroform and diluted with 1 mL methanol.

Additional chemical synthesis details can be found in Supporting Information File 1.

### HDAC8 enzymatic activity assay

A fluorogenic assay (BPS Bioscience, catalog # 50008) was performed to evaluate the inhibition potential of the designed inhibitors. The assay was carried out using the supplier’s instructions. **TOI1**, **TOI2**, **TOI3-rev**, **TOI4**, and panobinostat were evaluated at concentrations of 100 µM, 10 µM, and 1 µM in a 96 well plate. Trichostatin A (TCA), which was used as an inhibitor control, was also evaluated at 100 µM. The blank consisted of HDAC assay buffer without the addition of inhibitor or HDAC8, while the positive control consisted of HDAC8 without inhibitor added. The substrate concentration was 5 µM, and HDAC8 concentration per well was 4 ng/µL. The reaction was initiated by addition of enzyme. The fluorogenic substrate was excited at 360 nm and the emission signal was detected at 460 nm using a Biotek Synergy HTX multimodal plate reader after incubation for 30 minutes.

### Preparation of the HDAC8 receptor for docking

The HDAC8 crystal structure (protein database pdb: 1W22) [32] was utilized as the docking receptor for all compounds. This receptor is crystalized as a dimer thus only the A chain was prepared for docking using Sylbyl-X 2.1. A protocol defining the regions of hydrogen donor, acceptor and hydrophobic character was created using the SFXC protocol [41–43]. The Zn^2+^ ion which is known to be essential in the hydroxamate class of HDAC inhibitors was included in the preparation of the receptor.

### Preparation of inhibitor compounds for docking

Compounds were drawn in ChemDraw and the converted for 2D to 3D using Marvin Sketch. The energy gradient optimization method was used to perform an initial minimization in Marvin Sketch. A full structural minimization was performed in UCSF Chimera [44] using conjugate gradient followed by steepest decent. In Sybyl-X multiple conformers of each potential inhibitor compound were created for docking analysis using the prep protocol Docking >1 parameter.

### Molecular docking of inhibitors in the HDAC8 receptor

The inhibitor candidates were docked into the receptor using the Surflex-Dock Geom (SFXC) protocol [41–43] to evaluate the binding affinity of the ligand for the HDAC8 receptor. The C-scoring method was used to calculate these binding affinities and binding scores are given in −log_10_(*K*_d_) values [45]. Docking simulations where ran considering conformers at pH 7 to simulate the physiological conditions where the pH is 7.4. Results were analyzed in both Sybyl-X and UCSF Chimera.

## Supporting Information

File 1 Experimental and analytical data.
