# Supplementary material for: Design and synthesis of diazine-based panobinostat analogues for HDAC8 inhibition
Source: Beilstein J Org Chem. 2020 Apr 7;16:628–37. doi: 10.3762/bjoc.16.59 (PMC7155894; doi:10.3762/bjoc.16.59)
Supplement: File 1 — Experimental and analytical data. [file Beilstein_J_Org_Chem-16-628-s001.pdf]

BEILSTEIN JOURNAL OF ORGANIC CHEMISTRY

Supporting Information

for

Design and synthesis of diazine-based panobinostat analogues for HDAC8 inhibition

Sivaraman Balasubramaniam, Sajith Vijayan, Liam V. Goldman, Xavier A. May, Kyra Dodson, Sweta Adhikari, Fatima Rivas, Davita L. Watkins and Shana V. Stoddard

Beilstein J. Org. Chem. **2020,** *16,* 628–637. doi:10.3762/bjoc.16.59

Experimental and analytical data

License and Terms: This is a supporting information file under the terms of the Creative Commons Attribution License (http://creativecommons.org/licenses/by/4.0). Please note that the reuse, redistribution and reproduction in particular requires that the authors and source are credited. The license is subject to the *Beilstein Journal of Organic Chemistry* terms and conditions: (https://www.beilstein-journals.org/bjoc)

Table of contents **Pages**

**1.**	General chemistry procedures	S2
**2.**	Table of reaction conditions for **16**	S2
**3.**	Chemical synthesis	S3–S11
**4.**	NMR spectra	S12–S30
**5.**	References	S31

1. General chemistry procedures

All reactions were carried out under an inert atmosphere, unless otherwise noted. Reactions and purifications were monitored by thin layer chromatography using Silica gel 60 F254 (pre-coated on aluminium sheet, 0.2 mm thickness, Merck). Chromatographic purification was performed with silica gel 60 (230–400 mesh, Merck), reversed phase C-18 silica gel.

2. Table of reaction conditions for 16

Table S1: Reaction conditions evaluated for the synthesis of compound **16**.

S.No	Catalyst (10 mol %)	Base (2.0 equiv)	Solvent system	Yield[a]
1	$Pd(PPh_3)_4$	K_2CO_3	dioxane (4.5 mL)/water (1.5 mL)	No reaction
2	$Pd(PPh_3)_4$	K_2CO_3	DMF (4.5 mL)/water (1.5 mL)	No reaction
3	$PdCl_2(PPh_3)_2$	K_2CO_3	dioxane (4.5 mL)/water (1.5 mL)	No reaction
4	$PdCl_2(PPh_3)_2$	Na_2CO_3	dioxane (4.5 mL)/water (1.5 mL)	No reaction
5	$PdCl_2(PPh_3)_2$	Cs_2CO_3	dioxane (4.5 mL)/water (1.5 mL)	No reaction
6	$PdCl_2(PPh_3)_2$	Na_2HPO_4	dioxane (4.5 mL)/water (1.5 mL)	35 %
7	$PdCl_2(PPh_3)_2$	Na_2HPO_4	dioxane (4.5 mL)/water (1.5 mL)	8 %

[a]All reactions were repeated at least twice to ensure reproducibility.

3. Chemical synthesis

Methyl 5-methylpyrazine-2-carboxylate (5)[1]

To the starting material 5-methylpyrazine-2-carboxylic acid (2.0 g 0.145 mmol), methanol (20.0 mL), sulfuric acid (0.2 mL) and molecular sieves 4 Å (catalytic) was added and the reaction mixture was heated to reflux for 8 h. TLC revealed complete consumption of starting material after 8 h. The solvent was evaporated, EtOAC (25 mL) was added and water (10 mL) was added and the layers were separated. The aqueous layer was extracted twice with EtOAC (25 mL) and organic layers were pooled together, washed with brine solution (25 mL). The organic layer was dried over sodium sulfate and evaporated under reduced pressure to dryness. The compound was purified and washed using diethyl ether (10 mL). Yield: 81%, TLC: 30% EtOAc: 70% hexanes Rf: 0.3; ^{1}H NMR (500 MHz, $CDCl_3$) δ 9.2 (s, 1H), 8.54 (s, 1H), 4.03 (s, 3H), 2.67 (s, 3H).

5-Methylpyrazine-2-carbaldehyde (6)[2]

To the starting material methyl 5-methylpyrazine-2-carboxylate (500 mg 3.3 mmol), THF was added and this was cooled to −78 °C, followed by the addition of a 1.0 M methylene chloride solution of DIBAL-H (590 mg 3.6 mmol) and the mixture was stirred for 6 h at −78 °C. TLC revealed very little starting material (>5%). The reaction mixture was removed from the dry ice bath and was quenched as per Fieser workup. Then 0.14 mL of water was slowly added followed by the addition of ethyl acetate 15 mL, add 0.14 mL 15% aqueous sodium hydroxide. Then 0.36 mL water was added, warmed to rt and stirred for 15 min after addition of Na_2SO_4 (250 mg). The reaction mixture was filtered over Hyflo® bed and the bed was washed thoroughly with EtOAc (50 mL). The organic layer was evaporated under reduced pressure to dryness and purified using silica-gel flash chromatography. The pure product was characterized by NMR. Yield: 78%, TLC: 25%

EtOAc: 75% hexanes Rf: 0.35, ^{1}H NMR (500 MHz, $CDCl_3$) δ 10.12 (s, 1H), 9.05 (s, 1H), 8.62 (s, 1H), 2.69 (s, 3H).

Ethyl (*E*)-3-(5-methylpyrazin-2-yl)acrylate (7)

To the starting material 5-methylpyrazine-2-carbaldehyde (100 mg, 0.8 mmol), THF (1.0 mL) was added followed by phosphorene **8** (250 mg 1.7 mmol) at room temperature. The reaction mixture was stirred for 8 h at 60 °C. TLC revealed complete consumption of starting material. The reaction mixture was removed from heating bath and quenched using a saturated aqueous NH_4Cl solution. Water (10 mL) and ethyl acetate (25 mL) were added, layers were separated washed with brine solution, dried over sodium sulfate and evaporated under reduced pressure to dryness. Then column purification was done to get the pure product which was characterized by NMR. Yield: 72%, TLC: 25% EtOAc: 75% hexanes Rf: 0.35, white solid MP:114-118 °C, ^{1}H NMR (500 MHz, $CDCl_3$) δ 8.54 (s, 1H), 8.48 (s, 1H), 7.67 (d, J = 15.1 Hz, 1H), 6.96 (d, J = 15.3 Hz, 1H), 4.28 (s, 2H), 2.60 (s, 3H), 1.34 (s, 3H). ^{13}C NMR (126 MHz, $CDCl_3$) δ 166.3, 154.7, 145.9, 144.74, 143.8, 139.5, 123.4, 77.3, 77.1, 76.8, 60.8, 21.6, 14.2. HRMS (ESI-TOF) calculated for $C_{10}H_{13}N_2O_2$ ([M + H]$^+$): 193.0972, found: 193.0971. IR (neat, cm−1): 2866, 1738, 1552, 1402, 1304.

Ethyl (*E*)-3-(5-formylpyrazin-2-yl)acrylate (9)

To the starting material of ethyl (*E*)-3-(5-methylpyrazin-2-yl)acrylate **7** (25 mg 0.13 mmol), dioxane was added followed by SeO_2 (17 mg 0.15 mmol) at room temperature and was heated at 110 °C for 8 h. TLC revealed complete consumption of starting material. Ethyl acetate was added to the reaction mixture and the reaction mixture was filtered and evaporated to dryness under reduced pressure. Then column purification was done to get the pure product. The product was characterized by NMR. Yield: 61%, TLC: 35% EtOAc: 65% hexanes; Rf: 0.35, white solid MP: 156-158 °C, ^{1}H NMR (300 MHz, $CDCl_3$) δ 10.19

(s, 1H), 9.20 (s, 1H), 8.83 (s, 1H), 7.78 (d, *J* = 15.8 Hz, 1H), 7.20 (d, *J* = 15.8 Hz, 1H), 4.33 (t, *J* = 7.2 Hz, 2H), 1.39 (t, *J* = 7.2 Hz, 3H). ^{13}C NMR (126 MHz, $CDCl_3$) δ 191.8, 165.7, 152.0, 146.4, 144.7, 143.4, 138.1, 127.6, 61.2, 14.2. HRMS (ESI-TOF) calculated for $C_{10}H_{11}N_2O_3$ ([M + H]$^+$): 207.0761, found: 207.0759. IR (neat, cm−1): 2866, 1722, 1692, 1574, 1419, 1354, 1149

General procedure for Suzuki couplings

To the stirred solution of the halogenated compound (0.39 mmol) in dioxane (10 mL), $PdCl_2(PPh_3)_2$ (0.04 mmol), saturated solution of $NaHPO_4$ (2 mL, 1.2 mmol) were added and degassed using argon for 15 min. To this mixture, boronic acid (0.42 mmol) was added and degassing was continued for 15 more minutes following this TEA (4.0 equiv) was added to the turbid solution and degassed for additional 15 min. The reaction mixture was heated at 95 °C for 15 h. TLC revealed complete consumption of starting material. The reaction mixture was filtered using Hyflo® bed and the bed was washed thoroughly by ethyl acetate. The solvent mixture was evaporated under reduced pressure, and crude compound was loaded on a column and eluted using the mixture of ethyl acetate and hexanes. The products were characterized by NMR.

Methyl (*E*)-3-(5-methylpyrimidin-2-yl)acrylate (16)

Yield: 55%, TLC: 30% EtOAc: 70% hexanes, Rf: 0.25, white solid MP: 106-109 °C, ^{1}H NMR (500 MHz, $CDCl_3$) δ 8.80 (s, 2H), 7.63 (d, *J* = 16.2 Hz, 1H), 6.57 (d, *J* = 16.2 Hz, 1H), 3.86 (s, 3H), 2.80 (s, 3H). ^{13}C NMR (126 MHz, $CDCl_3$) δ 169.2, 166.2, 155.7, 137.7, 125.1, 120.6, 51.9, 25.9. HRMS (ESI-TOF) calculated for $C_9H_{11}N_2O_2$ ([M + H]$^+$): 179.0815, found: 179.0818. IR (neat, cm−1): 2877, 1705, 1574, 1441, 1324.

Methyl (*E*)-3-(5-formylpyrimidin-2-yl)acrylate (17)

Yield: 40%, TLC: 40% EtOAc: 60% hexanes Rf: 0.20, white solid MP: 194-197 °C, ^{1}H NMR (500 MHz, $CDCl_3$) δ 10.15 (s, 1H), 9.12 (s, 2H), 7.71 (d, J = 16.2 Hz, 1H), 6.74 (d, J = 16.2 Hz, 1H), 3.88 (s, 3H). ^{13}C NMR (126 MHz, $CDCl_3$) δ 190.2, 165.6, 159.2, 156.6, 136.1, 130.1, 124.5, 52.3. HRMS (ESI-TOF) calculated for $C_9H_9N_2O_3$ ([M + H]$^+$): 193.0608, found: 193.0604. IR (neat, cm−1): 2890, 1722, 1705, 1552, 1423, 1354, 1149

Methyl (*E*)-3-(2-methylpyrimidin-5-yl)acrylate (18)

Yield: 71%, TLC: 30% EtOAc: 70% hexanes, Rf: 0.25, white solid MP: 110-113 °C, ^{1}H NMR (300 MHz, $CDCl_3$) δ 8.79 (s, 2H), 7.62 (d, J = 16.2 Hz, 1H), 6.57 (d, J = 16.2 Hz, 1H), 3.84 (s, 3H). ^{13}C NMR (126 MHz, $CDCl_3$) δ 169.1, 166.2, 155.7, 137.8, 125.0, 120.5, 51.9, 25.9. HRMS (ESI-TOF) calculated for $C_9H_{11}N_2O_2$ ([M + H]$^+$): 179.0815, found: 179.0815. IR (neat, cm−1): 2860, 1719, 1562, 1412, 1184.

Methyl (*E*)-3-(6-methylpyridazin-3-yl)acrylate (24)

Yield: 41%, TLC: 30% EtOAc: 70% hexanes, Rf: 0.20, white solid MP:119-122 °C, ^{1}H NMR (500 MHz, $CDCl_3$) δ 7.88 (d, J = 16.0 Hz, 1H), 7.54 (d, J = 8.6 Hz, 1H), 7.38 (d, J = 8.6 Hz, 1H), 6.98 (d, J = 16.0 Hz, 1H), 3.87 (s, 3H), 2.79 (s, 3H). ^{13}C NMR (126 MHz, $CDCl_3$) δ 166.6, 160.0, 153.8, 140.9, 126.9, 125.5, 123.3, 52.1, 22.3. HRMS (ESI-TOF) calculated for $C_9H_{11}N_2O_2$ ([M + H]$^+$): 179.0815, found: 179.0816. IR (neat, cm−1): 2882, 1725, 1554, 1433, 1254.

General procedure for SeO_2 reaction

To the stirred solution of the starting material **7**, **18** or **24** (0.12 mmol) in dioxane (3mL), 0.24 mmol SeO_2 was added. The reaction mixture was heated at 110 °C in a sealed vial for 8 h for starting material **7**, **12** h for starting materials **18** and **24**. TLC revealed complete consumption of starting material. Ethyl acetate was added to the reaction mixture and the

reaction mixture was filtered, evaporated under reduced pressure and crude compound was purified using silica-gel flash chromatography and analyzed by NMR.

Methyl (*E*)-3-(2-formylpyrimidin-5-yl)acrylate (19)

Yield: 54%, TLC: 30% EtOAc: 70% hexanes, white solid MP:169-172 °C, ^{1}H NMR (500 MHz, $CDCl_3$) δ 10.15 (s, 1H), 9.12 (s, 2H), 7.71 (d, J = 16.2 Hz, 1H), 6.74 (d, J = 16.2 Hz, 1H), 3.88 (s, 3H). ^{13}C NMR (126 MHz, CDCl3) δ 190.3, 165.7, 159.3, 156.7, 136.2, 130.2, 124.5, 52.4. HRMS (ESI-TOF) calculated for $C_9H_9N_2O_3$ ([M + H]$^+$): 193.0608, found: 193.0606. IR (neat, cm−1): 2992, 1724, 1712, 1592, 1504, 1456, 1149.

Methyl (*E*)-3-(6-formylpyridazin-3-yl)acrylate (25)

Yield: 52%, TLC: 30% EtOAc: 70% hexanes, white solid MP:164-166 °C, ^{1}H NMR (500 MHz, $CDCl_3$) δ 10.44 (s, 1H), 8.09 (d, J = 8.6 Hz, 1H), 7.92 (d, J = 16.0 Hz, 1H), 7.82-7.77 (m, 1H), 7.21 (d, J = 16.0 Hz, 1H), 3.90 (s, 3H). ^{13C} NMR (126 MHz, $CDCl_3$) δ 191.6, 165.9, 157.9, 154.5, 139.3, 126.9, 126.5, 124.6, 52.3. HRMS (ESI-TOF) calculated for $C_9H_9N_2O_3$ ([M + H]$^+$): 193.0608, found: 193.0604. IR (neat, cm−1): 2989, 1721, 1709, 1584, 1532, 1441, 1134.

General procedure for the reductive amination reaction

To the stirred solution/suspension of aldehyde (0.3 mmol) and indole amine (0.36 mmol) in dichloroethane (1.8 mL), sodium triacetoxyhydroborate (STAB) (0.33 mmol) followed by triethylamine (0.25 mL) was added at room temperature and stirred for 15 h. TLC revealed complete consumption of starting materials. Water and chloroform was added, and layers were separated. The organic layer was dried over sodium sulfate, evaporated under reduced pressure to dryness and crude compound was purified using silica-gel flash chromatography. The pure product was characterized by NMR.

Ethyl (*E*)-3-(5-(((2-(2-methyl-1*H*-indol-3-yl)ethyl)amino)methyl)pyrazin-2-yl)acrylate (11)

Yield: 63%. TLC: 3% MeOH: 97% DCM; Rf: 0.15, brown solid MP:108-111 °C, ^{1}H NMR (500 MHz, Methanol-d4) *δ* 8.67 (s, 1H), 8.63 (s, 1H), 7.73 (d, *J* = 15.8 Hz, 1H), 7.40 (d, *J* = 7.8 Hz, 1H), 7.26 (d, *J* = 8.0 Hz, 1H), 7.05-7.00 (m, 2H), 6.94-6.91 (m, 1H), 4.29 (t, *J* = 7.2 Hz, 2H), 3.09-3.06 (m, 4H), 2.41 (s, 3H), 1.36 (t, *J* = 7.1 Hz, 3H). 13C NMR (126 MHz, Methanol-d4) *δ* 164.7, 150.6, 145.9, 142.3, 137.7, 134.3, 130.7, 126.6, 122.3, 118.6, 116.7, 115.2, 110.0, 108.5, 104.4, 59.1, 48.4, 21.0, 11.6, 8.4. HRMS (ESI-TOF) calculated for $C_{21}H_{25}N_4O_2$ ($[M + H]^+$): 365.1972, found: 365.1986. IR (neat, cm−1): 3092, 1705, 1574, 1552, 1402, 1354, 1149

Methyl (*E*)-3-(5-(((2-(2-methyl-1*H*-indol-3-yl)ethyl)amino)methyl)pyrimidin-2-yl)acrylate (22)

Yield: 61%, TLC: Rf: 0.25 (10% MeOH: 90% DCM); ^{1}H NMR (500 MHz, $CDCl_3$) *δ* 8.68 (s, 2H), 7.81 (s, 1H), 7.54 (d, *J* = 16.2 Hz, 1H), 7.42 (d, *J* = 7.7 Hz, 1H), 7.25-7.22 (m, 1H), 7.02 (m, 3H), 6.49 (d, *J* = 16.2 Hz, 1H), 4.07 (s, 2H), 3.80 (s, 3H), 2.99-2.85 (m, 4H), 2.37 (s, 3H). ^{13}C NMR (126 MHz, MeOD) *δ* 166.7, 165.0, 154.2, 135.8, 134.2, 130.4, 126.8, 124.9, 119.5, 118.4, 116.4, 115.5, 108.4, 105.6, 52.3, 49.5, 47.2, 22.0, 8.5. HRMS (ESI-TOF) calculated for $C_{20}H_{23}N_4O_2$ ($[M + H]^+$): 351.1816, found: 351.1829. IR (neat, cm−1): 2866, 1705, 1574, 1552, 1402, 1354, 1149

Methyl-(*E*)-3-(2-(((2-(2-methyl-1*H*-indol-3-yl)ethyl)amino)methyl)pyrimidin-5-yl)acrylate (23)

Yield: 68%, TLC: Rf: 0.25 (10% MeOH: 90% DCM); ^{1}H NMR (500 MHz, $CDCl_3$) *δ* 8.74 (s, 2H), 7.92 (s, 1H), 7.60 (d, *J* = 16.2 Hz, 1H), 7.48-7.45 (m, 1H), 7.28 (d, *J* = 1.5 Hz, 1H), 7.17 -7.09 (m, 1H), 7.09-6.96 (m, 1H), 6.55 (d, *J* = 16.2 Hz, 1H), 4.13 (s, 2H), 3.86

(s, 3H), 2.99 (m, 4H), 2.42 (s, 3H). ^{13}C NMR (126 MHz, $CDCl_3$) δ 169.5, 166.3, 155.6, 137.5, 135.6, 131.8, 128.6, 125.9, 121.0, 120.8, 119.0, 117.9, 110.2, 108.8, 55.1, 52.0, 49.6, 29.7, 24.7, 11.7. HRMS (ESI-TOF) calculated for $C_{20}H_{23}N_4O_2$ ($[M + H]^+$): 351.1816, found: 351.1827. IR (neat, cm−1): 2866, 1705, 1574, 1552, 1402, 1354, 1149.

Methyl (*E*)-3-(6-(((2-(2-methyl-1*H*-indol-3-yl)ethyl)amino)methyl)pyridazin-3-yl)acrylate (26)

Yield: 38%, TLC: Rf: 0.25 (10% MeOH: 90% DCM); ^{1}H NMR (500 MHz, Methanol-d_4) δ 7.97 (d, *J* = 8.7 Hz, 1H), 7.86 (d, *J* = 16.1 Hz, 1H), 7.68 (d, *J* = 8.7 Hz, 1H), 7.43 (d, *J* = 7.8 Hz, 1H), 7.26 (m, 1H), 6.99-6.91 (m, 3H), 4.37 (s, 2H), 3.86 (s, 3H), 3.17-3.12 (m, 4H), 2.42 (s, 3H). ^{13}C NMR (126 MHz, MeOD) δ 166.3, 158.2, 155.7, 139.9, 135.8, 132.7, 128.1, 127.1, 126.5, 124.4, 120.2, 118.3, 116.8, 110.1, 105.8, 70.2, 51.2, 50.5, 22.3, 9.9. HRMS (ESI-TOF) calculated for $C_{20}H_{23}N_4O_2$ ($[M + H]^+$): 351.1816, found: 351.1824. IR (neat, cm−1): 289, 1705, 1574, 1552, 1402, 1354, 1149.

General procedure for hydroxamic acid synthesis

To the stirred solution of the starting material ethyl/methyl ester (0.02 mmol) in methanol (1.0 mL) at 0 °C, NaOH (0.75 mmol) in methanol (1.0 mL) was added at −10 °C followed by the addition of 50% aqueous hydroxylamine solution (0.3 mL, 0.02 mmol) at −10 °C and gradually allowed to attain room temperature and stirred for 12 h. TLC revealed complete consumption of starting material. Saturated ammonium chloride solution was added and the reaction mixture was evaporated under reduced pressure and azeotrope with methanol to remove water. C18 column was performed. After several trials with MeOH and DCM, water and ACN, water and THF, Water and MeOH. It was found pure compound was isolate by carrying out the column only in water and gradually increasing methanol. The compound got eluted in 10% MeOH: 90% water mixture.

(*E*)-*N*-Hydroxy-3-(5-(((2-(2-methyl-1*H*-indol-3-yl)ethyl)amino)methyl)pyrazin-2-yl)acrylamide (TOI1)

Yield: 55%, Rf: 0.10 (15% MeOH: 85% DCM), brown solid MP:158-161 °C ^{1}H NMR (500 MHz, Methanol-d_4) δ 8.54 (s, 1H), 8.52 (s, 1H), 7.56 (d, J = 15.5 Hz, 1H), 7.36 (d, J = 7.8 Hz, 1H), 7.23 (d, J = 8.0 Hz, 1H), 7.05 – 6.95 (m, 2H), 6.92-6.90 (m, 1H), 3.93 (s, 2H), 2.98-2.92 (m, 4H), 2.38 (s, 3H). ^{13}C NMR (126 MHz, Methanol-d4) δ 164.2, 149.4, 148.2, 144.6, 136.7, 135.6, 133.7, 128.7, 125.0, 121.3, 119.4, 117.5, 111.1, 105.0, 49.3, 49.1, 21.8, 10.8.HRMS (ESI-TOF) calculated for $C_{19}H_{22}N_5O_2$ ([M + H]$^+$): 352.1768, found: 352.1780. IR (neat, cm−1): 2996, 1701, 1574, 1434, 1149.

(*E*)-*N*-Hydroxy-3-(5-(((2-(2-methyl-1*H*-indol-3-yl)ethyl)amino)methyl)pyrimidin-2-yl)acrylamide (TOI2)

Yield: 49%, Rf: 0.12 (15% MeOH: 85% DCM), ^{1}H NMR (500 MHz, DMSO-d_6) δ 10.66 (s, 1H), 8.92 (s, 2H), 7.44 (d, J = 15.9 Hz, 1H), 7.36 (d, J = 7.8 Hz, 1H), 7.20 (d, J = 8.0 Hz, 1H), 6.95 (t, J = 7.6 Hz, 1H), 6.88 (t, J = 7.4 Hz, 1H), 6.63 (d, J = 16.0 Hz, 1H), 4.03 (s, 1H), 3.93 (s, 2H), 2.78-2.74 (m, 4H), 2.30 (s, 3H). ^{13}C NMR (126 MHz, MeOD) δ 164.8, 160.9, 152.7, 133.2, 129.6, 129.4, 125.7, 124.5, 118.7, 117.4, 115.4, 114.4, 107.3, 104.4, 51.2, 46.2, 20.9, 7.5. HRMS (ESI-TOF) calculated for $C_{19}H_{22}N_5O_2$ ([M + H]$^+$): 352.1768, found: 352.1774. IR (neat, cm−1): 3002, 1695, 1429, 1354.

(*E*)-*N*-Hydroxy-3-(2-(((2-(2-methyl-1*H*-indol-3-yl)ethyl)amino)methyl)pyrimidin-5-yl)acrylamide (TOI3-rev)

Yield: 51%, Rf: 0.12 (15% MeOH: 85% DCM); ^{1}H NMR (500 MHz, Methanol-d_4) δ 8.78 (s, 2H), 7.48 (d, J = 16.1 Hz, 1H), 7.35 (d, J = 7.9 Hz, 1H), 7.24 (d, J = 8.1 Hz, 1H), 7.00 (t, J = 7.5 Hz, 1H), 6.90 (t, J = 7.5 Hz, 1H), 6.62 (d, J = 15.9 Hz, 1H), 4.02 (s, 2H), 3.37-2.95 (m, 4H), 2.39 (s, 3H). ^{13}C NMR (126 MHz, MeOD) δ 165.8, 161.9, 153.8, 134.2,

130.6, 130.4, 126.7, 125.5, 119.7, 118.4, 116.7, 115.4, 108.3, 105.5, 52.1, 47.2, 21.9, 8.4. HRMS (ESI-TOF) calculated for $C_{19}H_{22}N_5O_2$ ([M + H]$^+$): 352.1768, found: 352.1769. IR (neat, cm−1): 2982, 1689, 1552, 1402, 1147.

(*E*)-*N*-Hydroxy-3-(6-(((2-(2-methyl-1*H*-indol-3-yl)ethyl)amino)methyl)pyridazin-3-yl)acrylamide (TOI4)

Yield: 44%, Rf: 0.12 (15% MeOH: 85% DCM); ^{1}H NMR (500 MHz, Methanol-*d*$_4$) δ 7.79 (d, *J* = 8.7 Hz, 1H), 7.72 (d, *J* = 15.8 Hz, 1H), 7.63 (d, *J* = 8.7 Hz, 1H), 7.38 (d, *J* = 7.9 Hz, 1H), 7.24 (d, *J* = 8.0 Hz, 1H), 7.05-6.97 (m, 2H), 6.93 (d, *J* = 7.6 Hz, 1H), 4.16 (s, 2H), 2.98-2.92 (m, 4H), 2.39 (s, 3H). ^{13}C NMR (126 MHz, MeOD) δ 163.2, 155.5, 135.8, 135.4, 128.3, 127.2, 126.5, 124.1, 120.0, 118.1, 116.2, 109.9, 51.6, 23.4, 9.9. HRMS (ESI-TOF) calculated for $C_{19}H_{22}N_5O_2$ ([M + H]$^+$): 352.1768, found: 352.1767. IR (neat, cm−1): 2991, 1689, 1519, 1426, 1314, 1150.

3-(5-(((2-(2-Methyl-1*H*-indol-3-yl)ethyl)amino)methyl)pyrazin-2-yl)isoxazolidin-5-one (12)

Yield: 40%, Rf: 0.20 (15% MeOH: 85% DCM); ^{1}H NMR (500 MHz, Methanol-*d*$_4$) δ 8.62 (s, 1H), 8.58 (s, 1H), 7.43 (d, *J* = 7.8 Hz, 1H), 7.26 (d, *J* = 8.0 Hz, 1H), 7.05-7.01 (m, 2H), 4.53 (t, *J* = 7.1 Hz, 1H), 4.10 (s, 2H), 3.09-3.01 (m, 4H), 2.66 (dd, *J* = 14.9, 7.1 Hz, 2H), 2.39 (s, 3H), 1.94 (s, 3H). ^{13}C NMR (126 MHz, MeOD) δ 168.7, 154.3, 151.9, 143.0, 143.4, 135.6, 132.6, 128.8, 120.8, 120.1, 118.1, 116.9, 110.0, 106.0, 61.4, 50.7, 48.6, 48.4, 34.4, 22.4, 10.1. HRMS (ESI-TOF) calculated for $C_{19}H_{22}N_5O_2$ ([M + H]$^+$): 352.1768, found: 352.1764. IR (neat, cm−1): 2910, 1725, 1501, 1402, 1354, 1149.

4. NMR Spectra

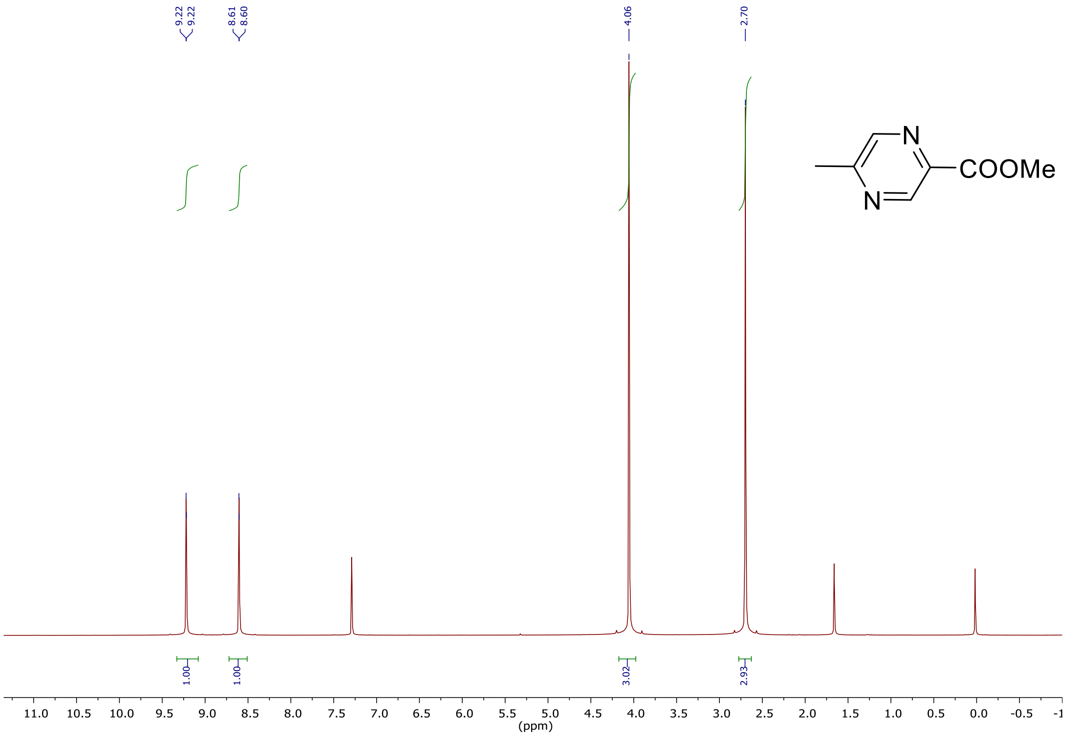

Figure S1. ^{13}C NMR spectrum of compound **5** ($CDCl_3$, 500 MHz).

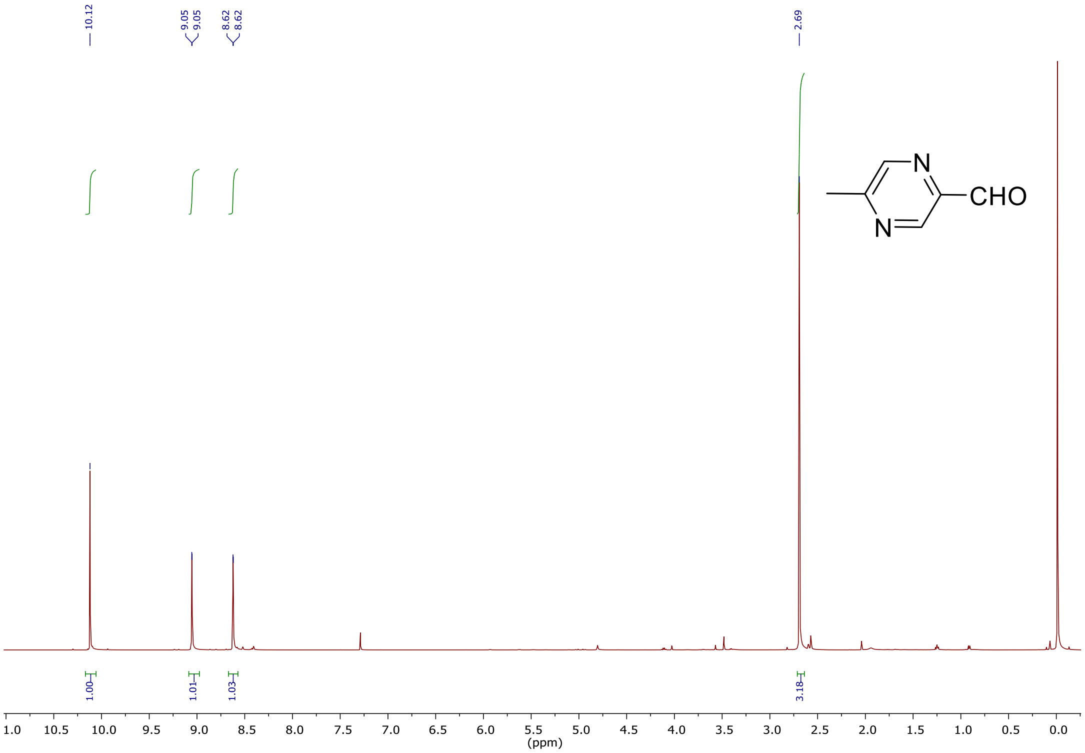

Figure S2. ^{1}H NMR spectrum of compound **6** ($CDCl_3$, 500 MHz).

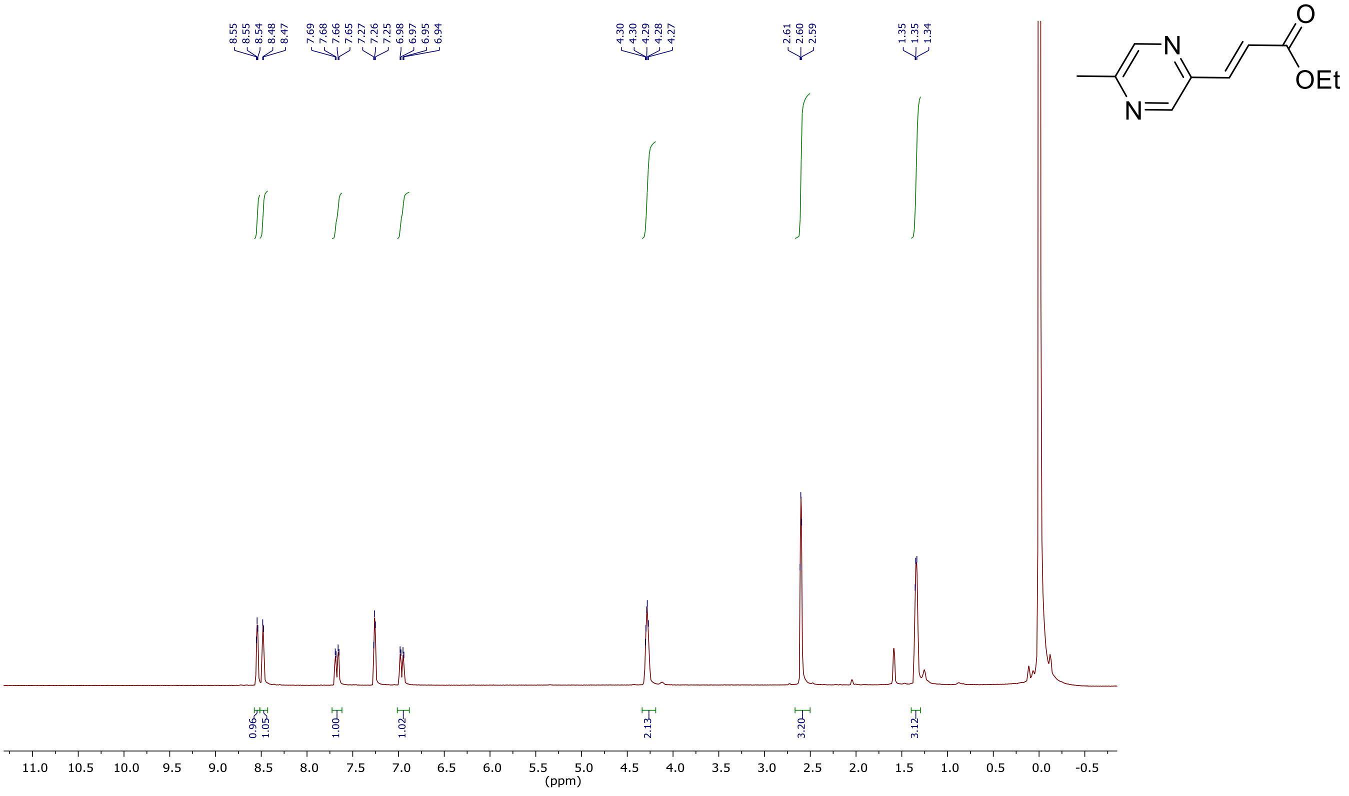

Figure S3. ^{1}H NMR spectrum of compound **7** ($CDCl_3$, 500 MHz).

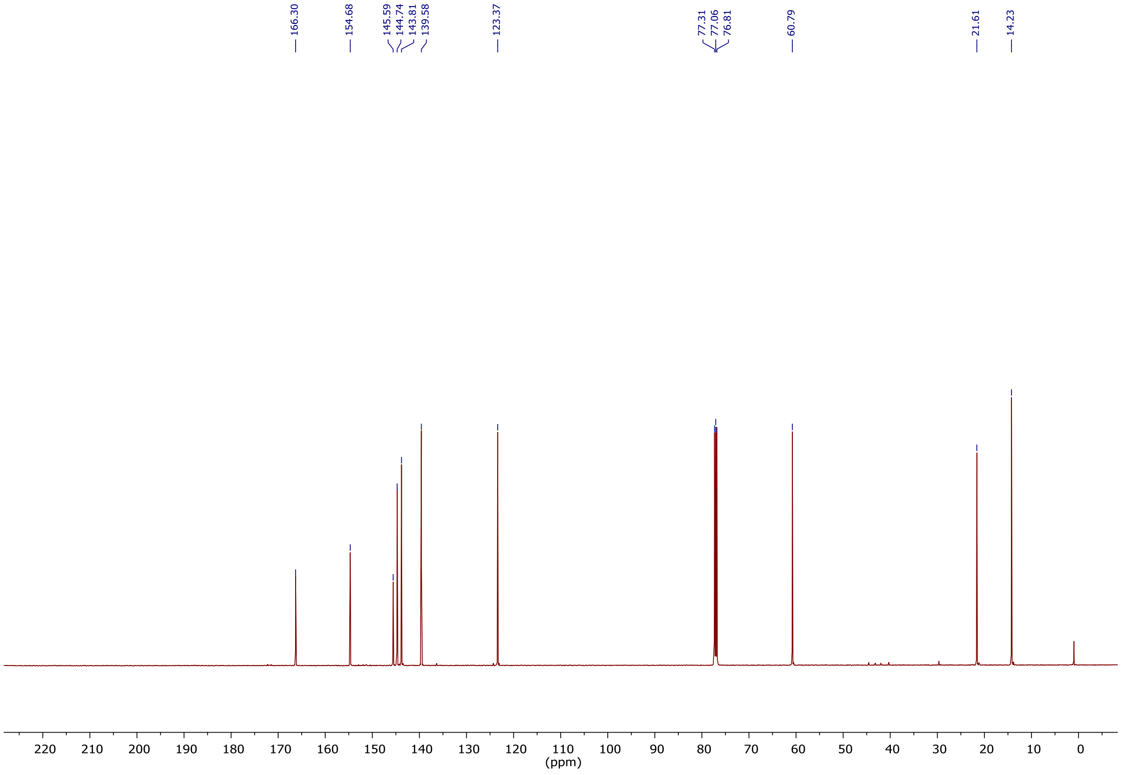

Figure S4. ^{13}C NMR spectrum of compound **7** ($CDCl_3$, 126 MHz).

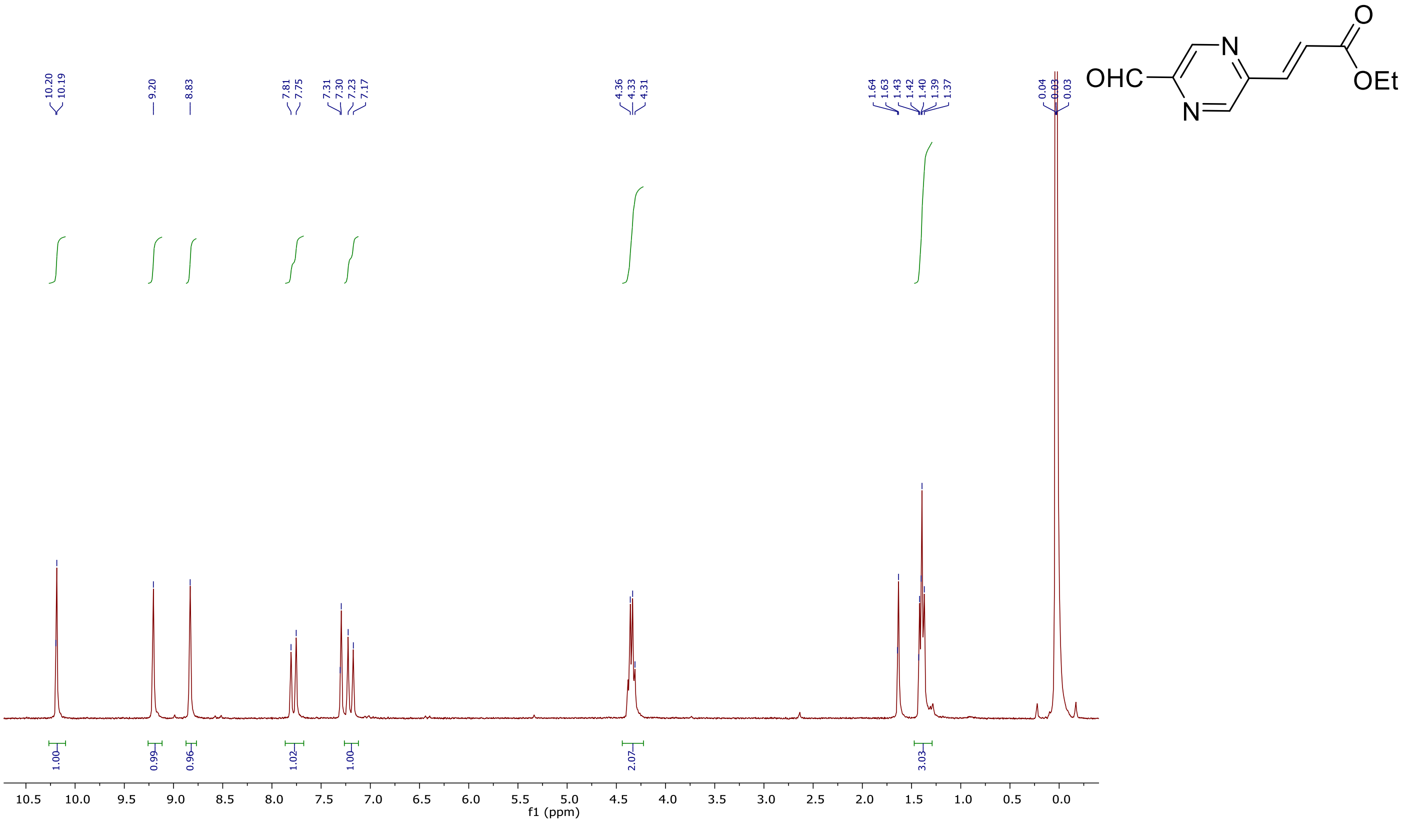

Figure S5. ^{1}H NMR spectrum of compound **9** ($CDCl_3$, 500 MHz).

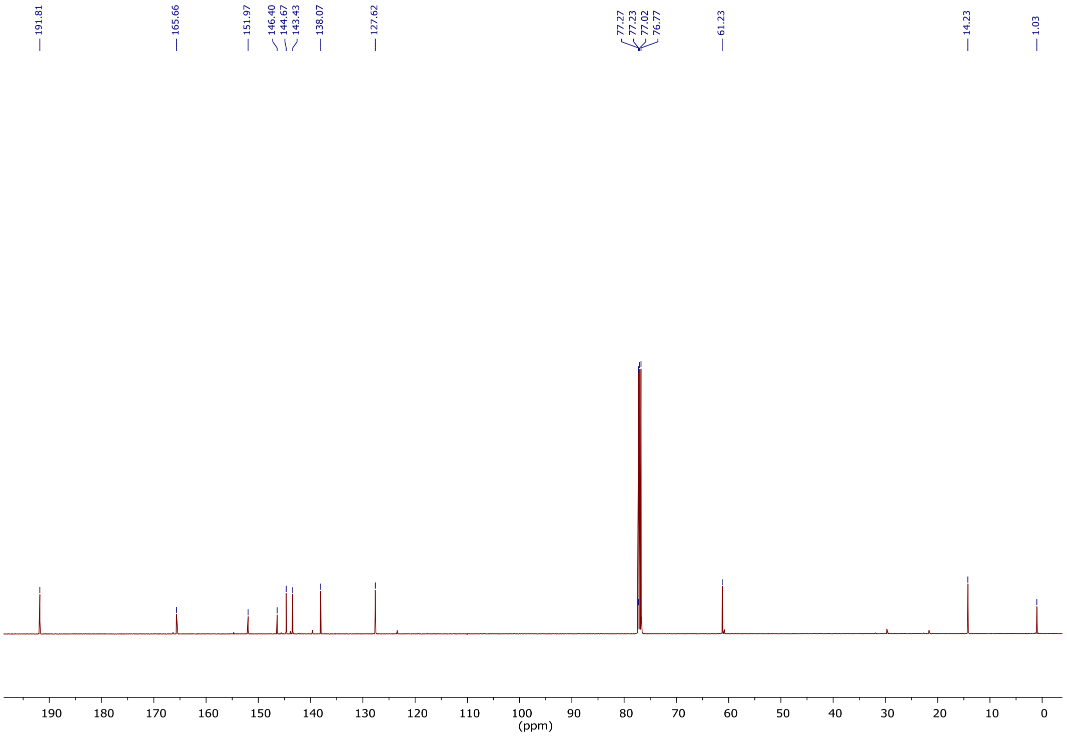

Figure S6. ^{13}C NMR spectrum of compound **9** ($CDCl_3$, 126 MHz).

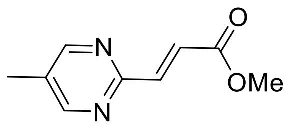

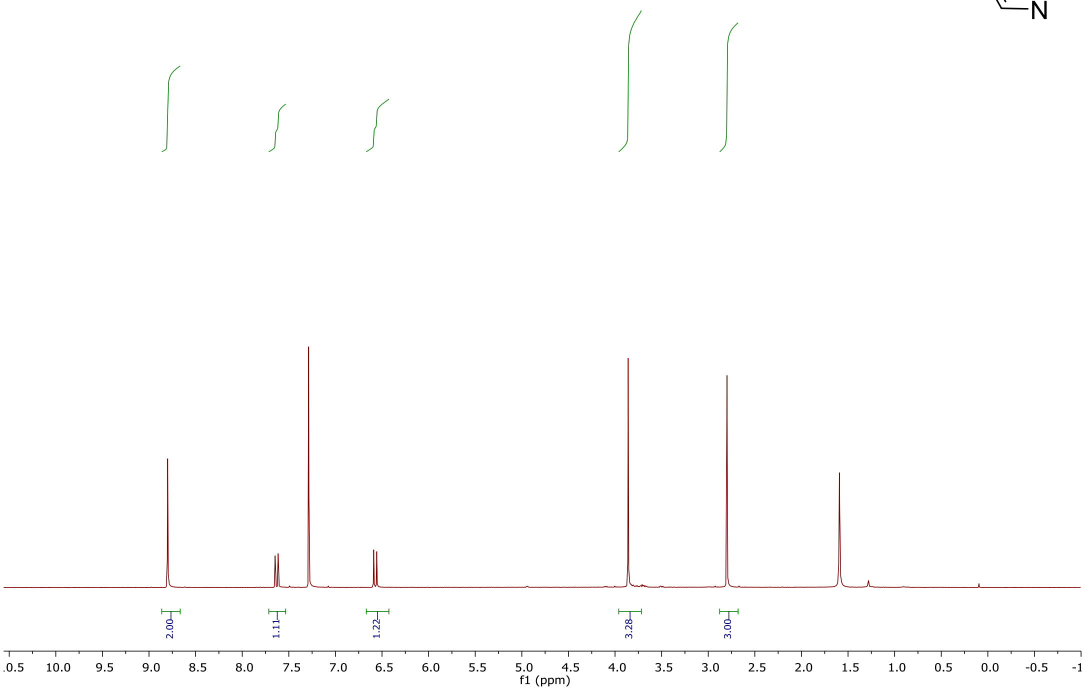

Figure S7. ^{1}H NMR spectrum of compound **16** ($CDCl_3$, 500 MHz).

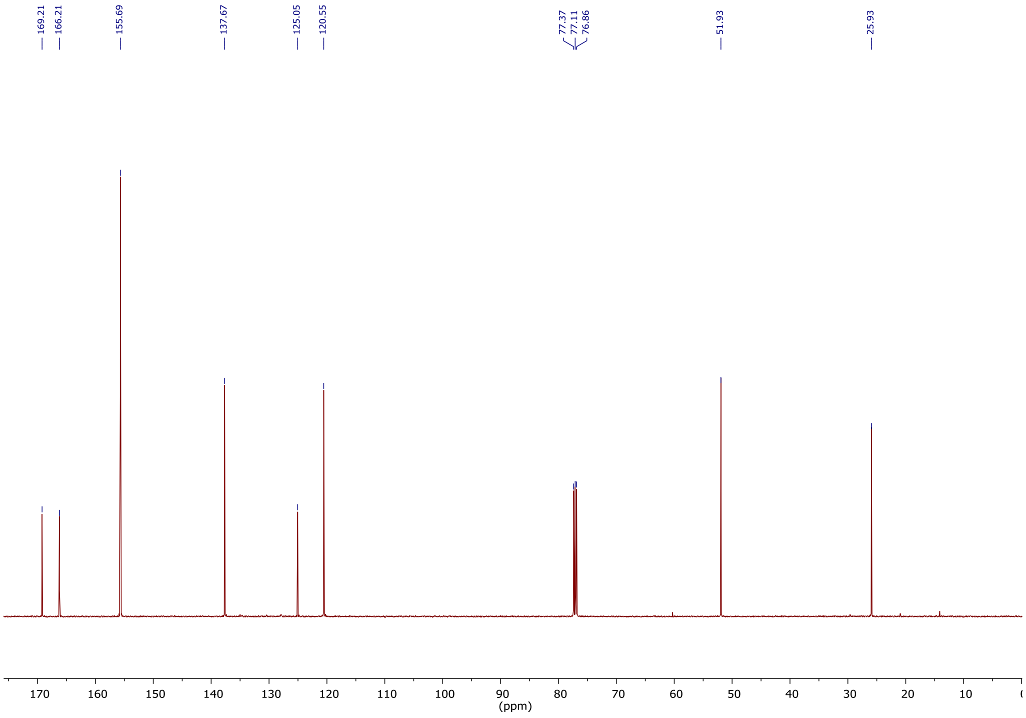

Figure S8. ^{13}C NMR spectrum of compound **16** ($CDCl_3$, 126 MHz).

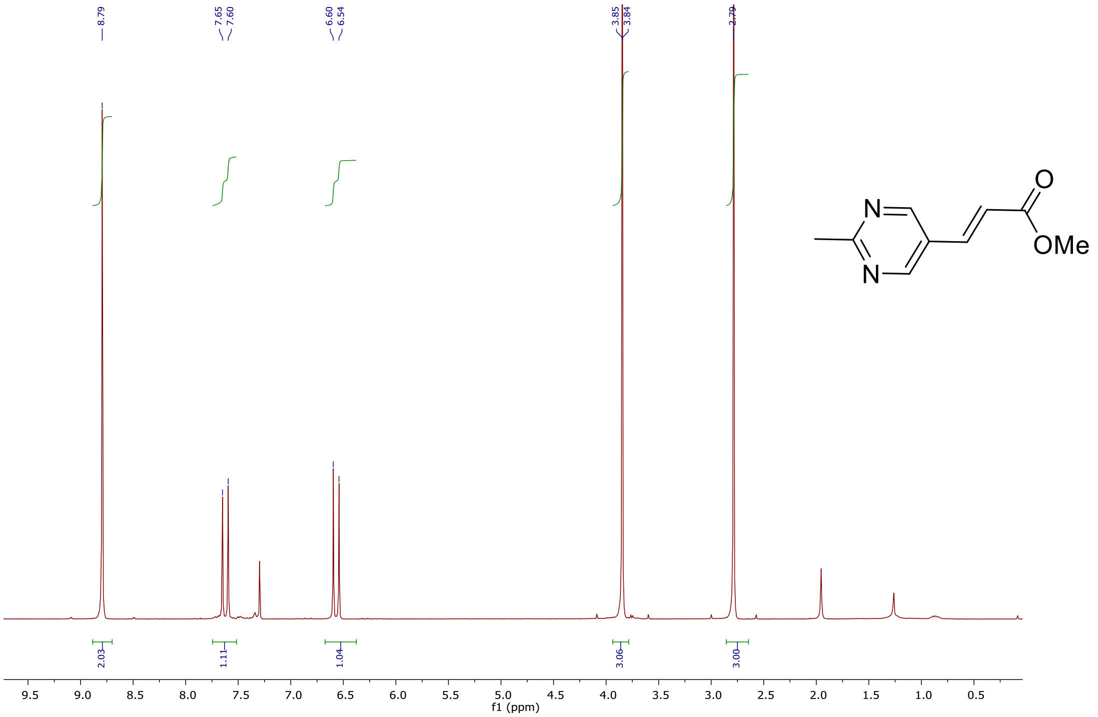

Figure S9. ^{1}H NMR spectrum of compound **18** ($CDCl_3$, 500 MHz).

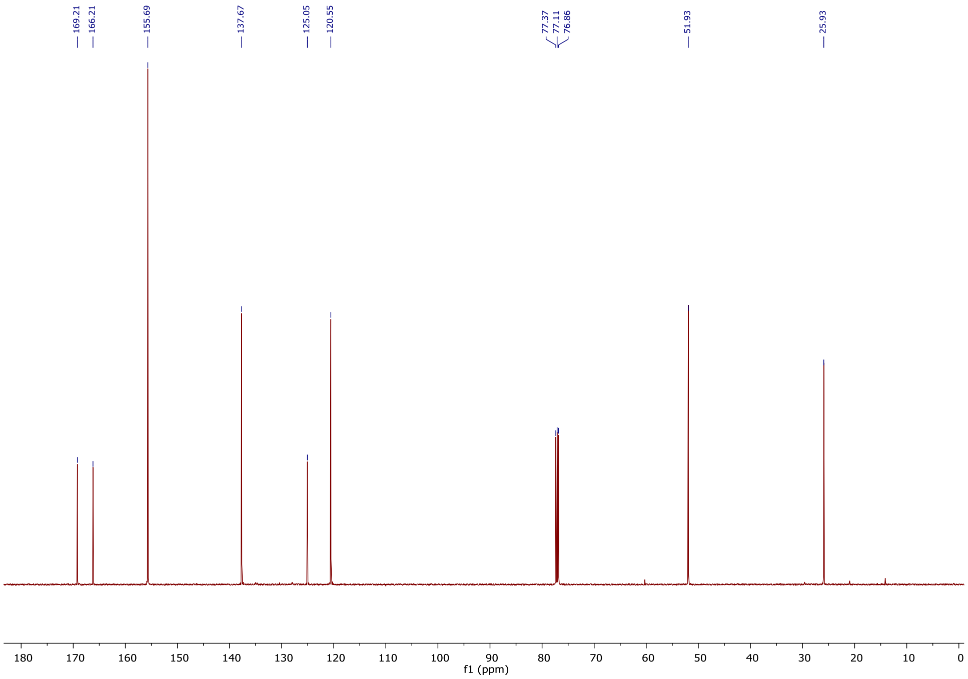

Figure S10. ^{13}C NMR spectrum of compound **18** ($CDCl_3$, 126 MHz).

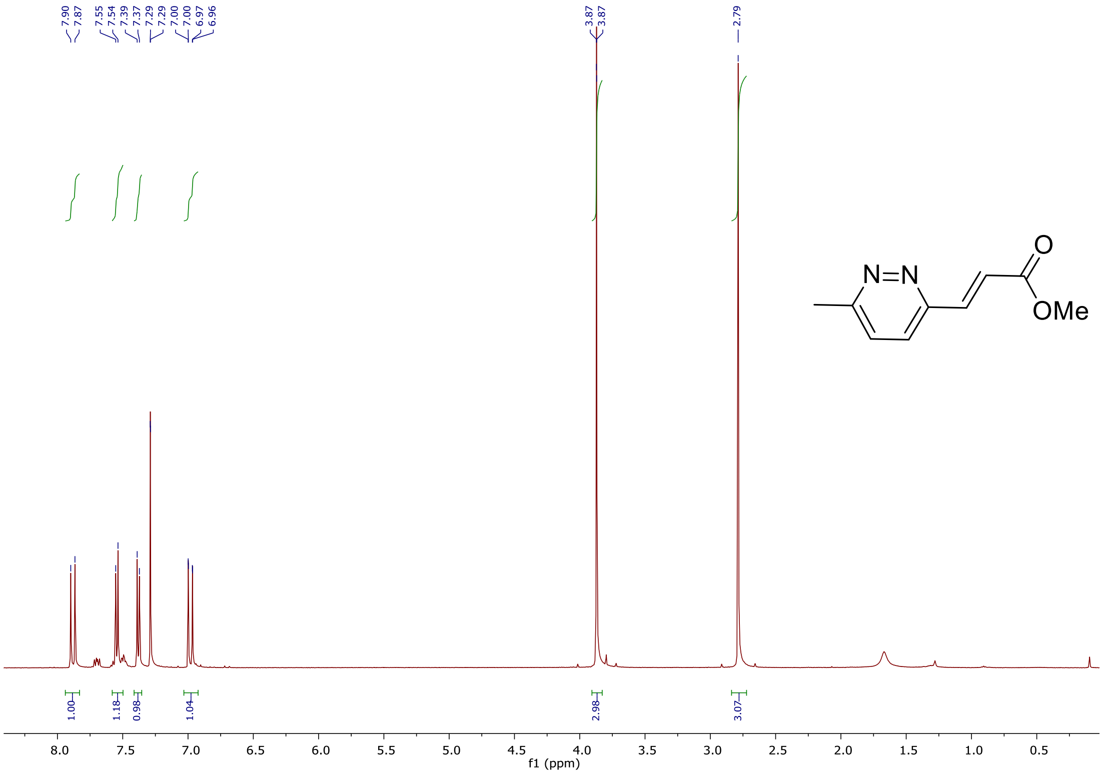

Figure S11. ^{1}H NMR spectrum of compound **24** ($CDCl_3$, 500 MHz).

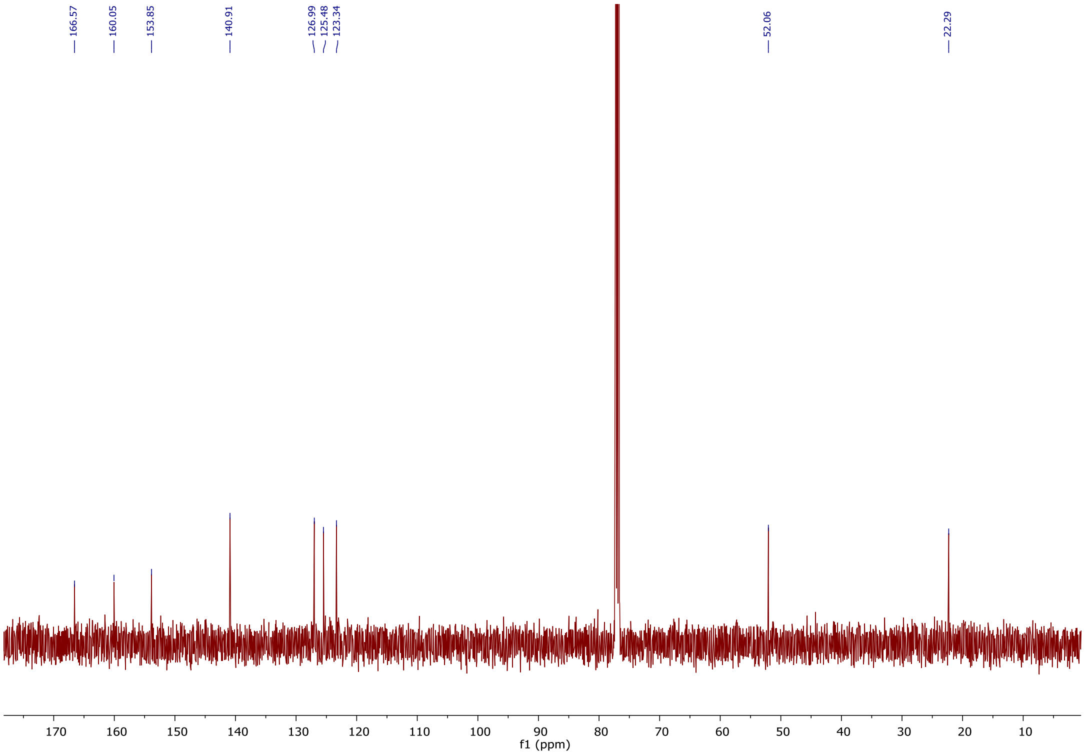

Figure S12. ^{13}C NMR spectrum of compound **24** ($CDCl_3$, 126 MHz).

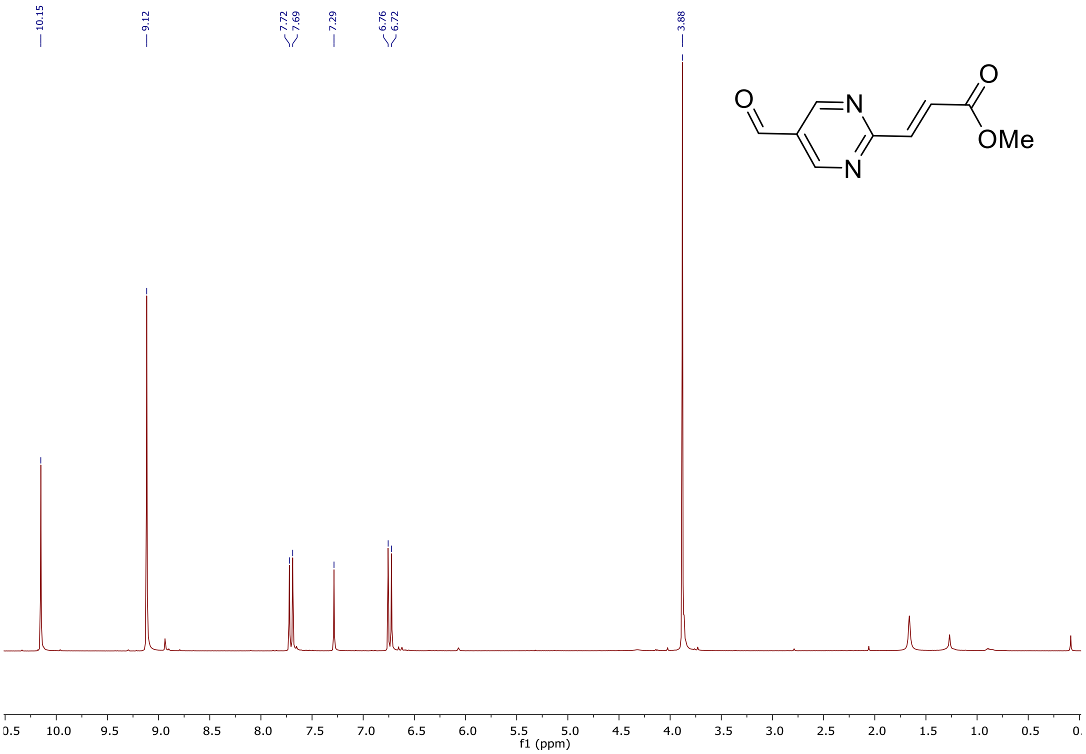

Figure S13. ^{1}H NMR spectrum of compound **17** ($CDCl_3$, 500 MHz).

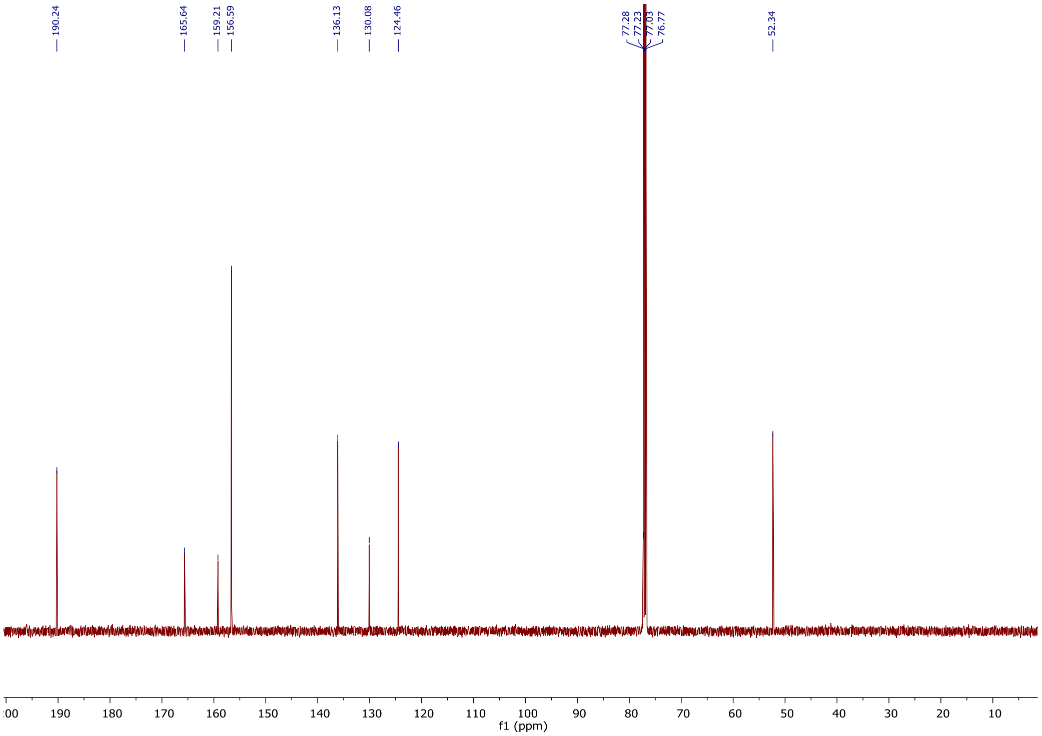

Figure S14. ^{13}C NMR spectrum of compound **17** ($CDCl_3$, 126 MHz).

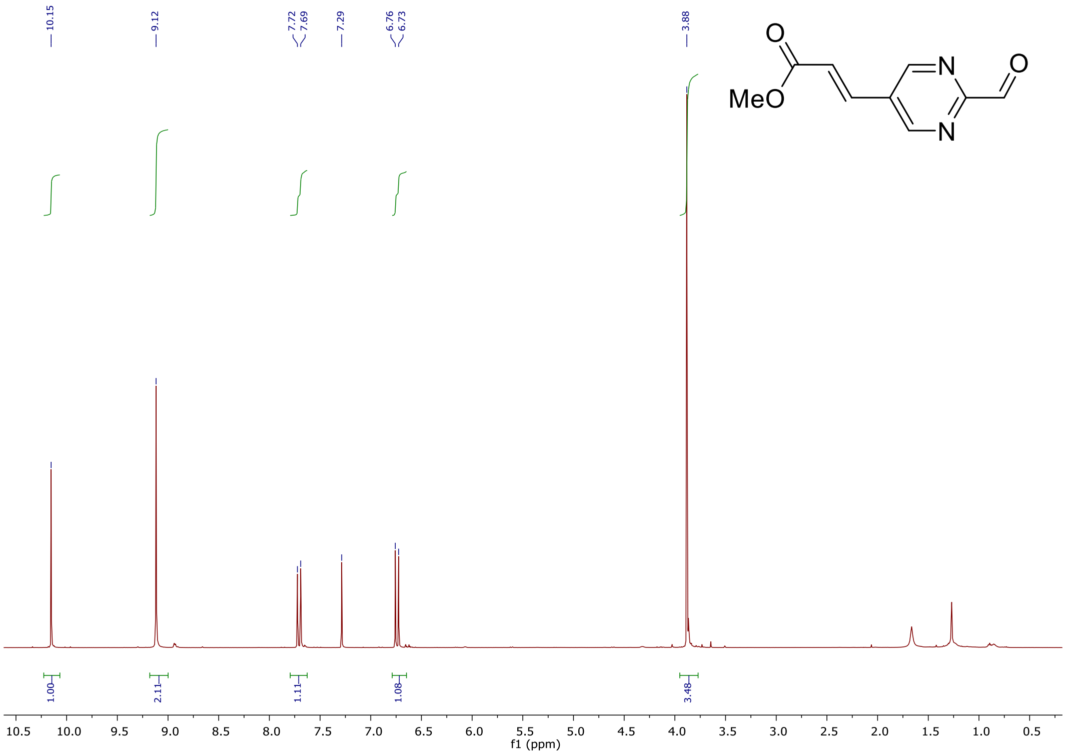

Figure S15. ^{1}H NMR spectrum of compound **19** ($CDCl_3$, 500 MHz).

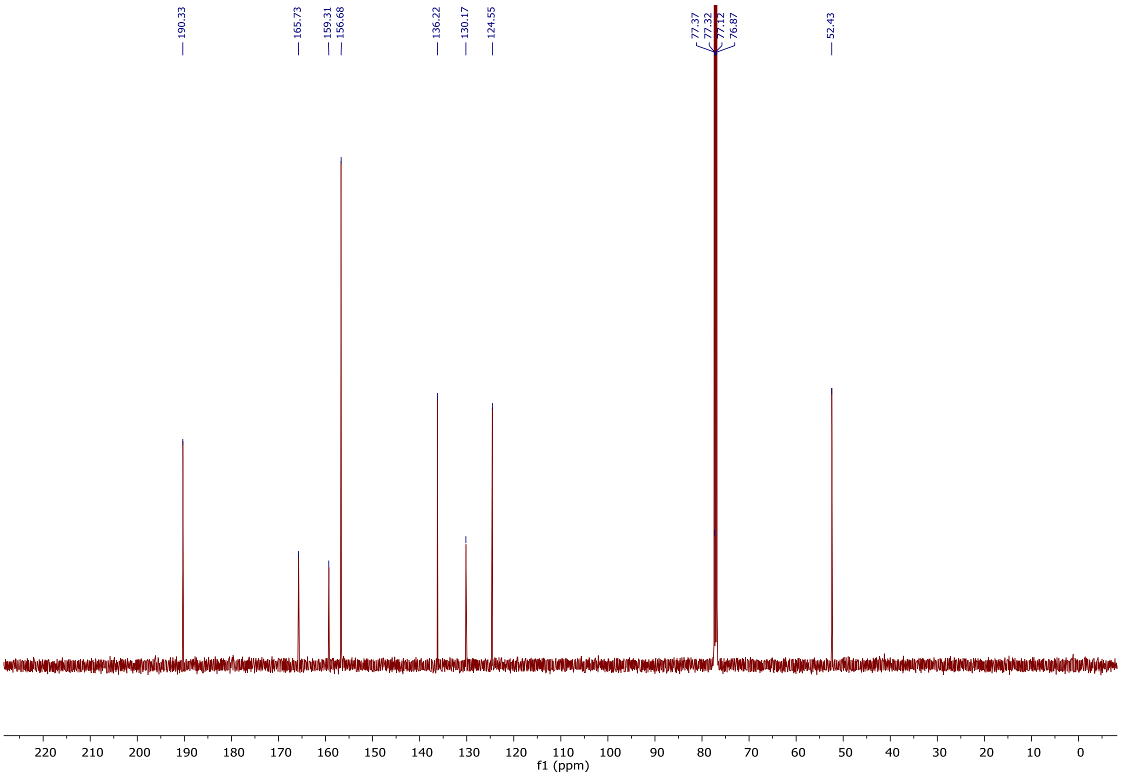

Figure S16. ^{13}C NMR spectrum of compound **19** ($CDCl_3$, 126 MHz).

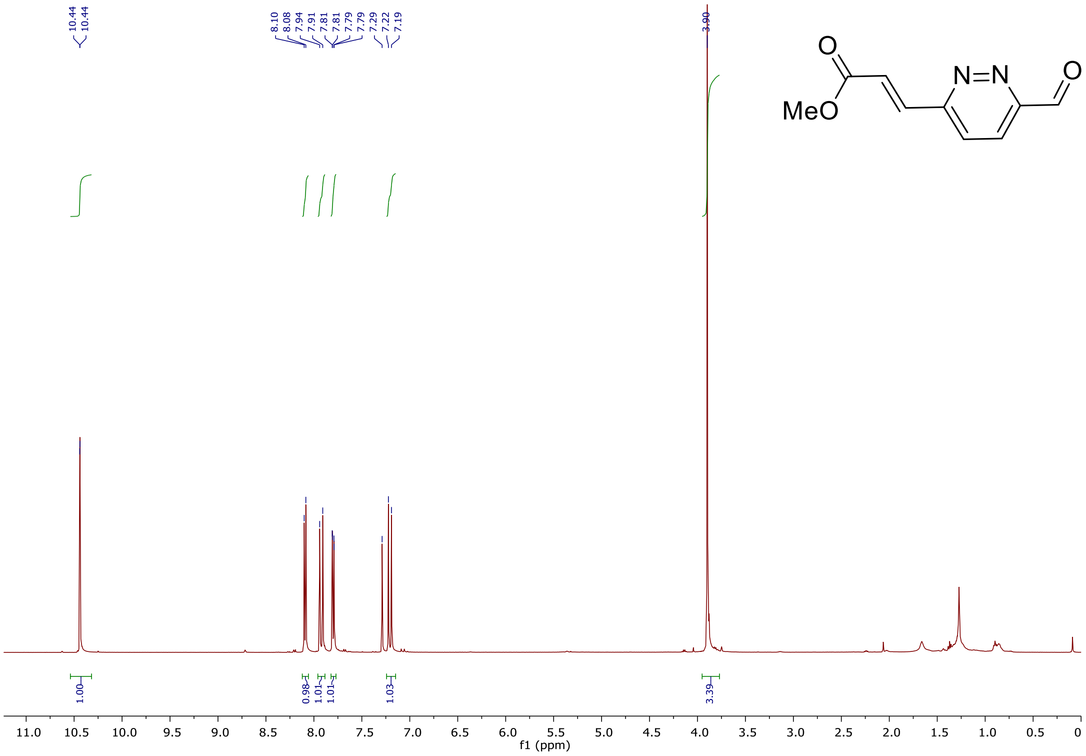

Figure S17. ^{1}H NMR spectrum of compound **25** ($CDCl_3$, 500 MHz).

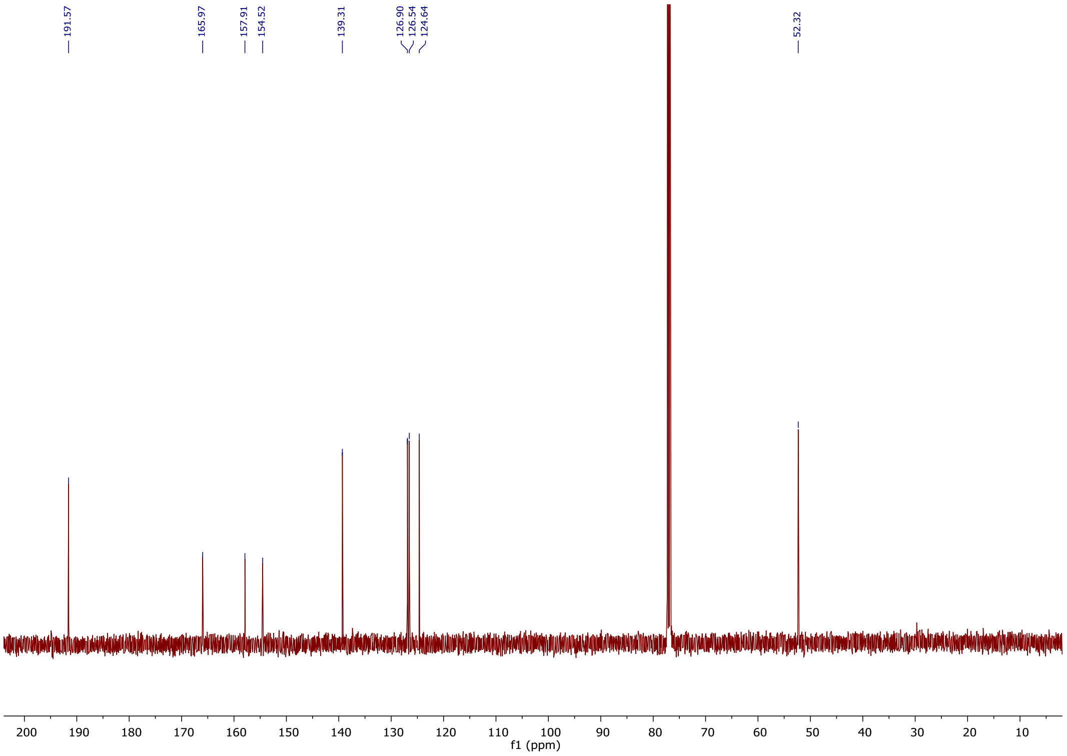

Figure S18. ^{13}C NMR spectrum of compound **25** ($CDCl_3$, 126 MHz).

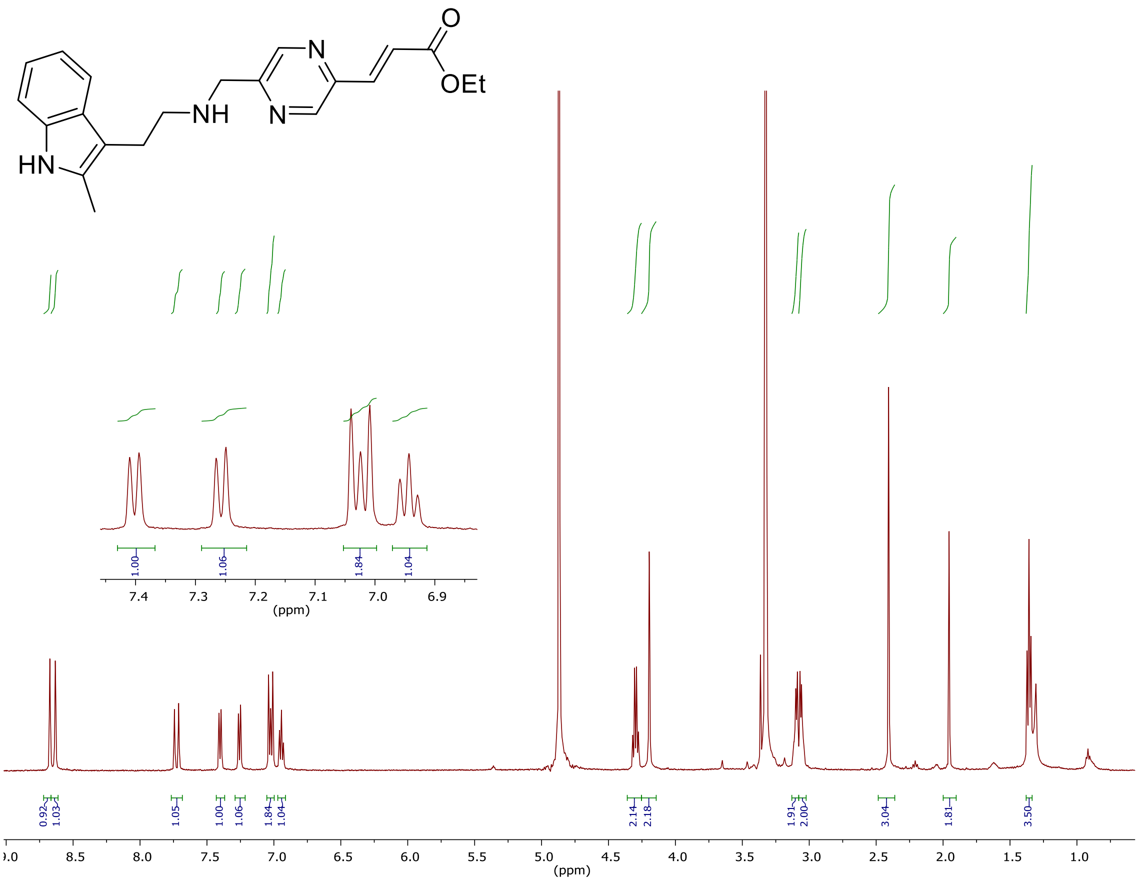

Figure S19. ^{1}H NMR spectrum of compound **11** (MeOD, 500 MHz).

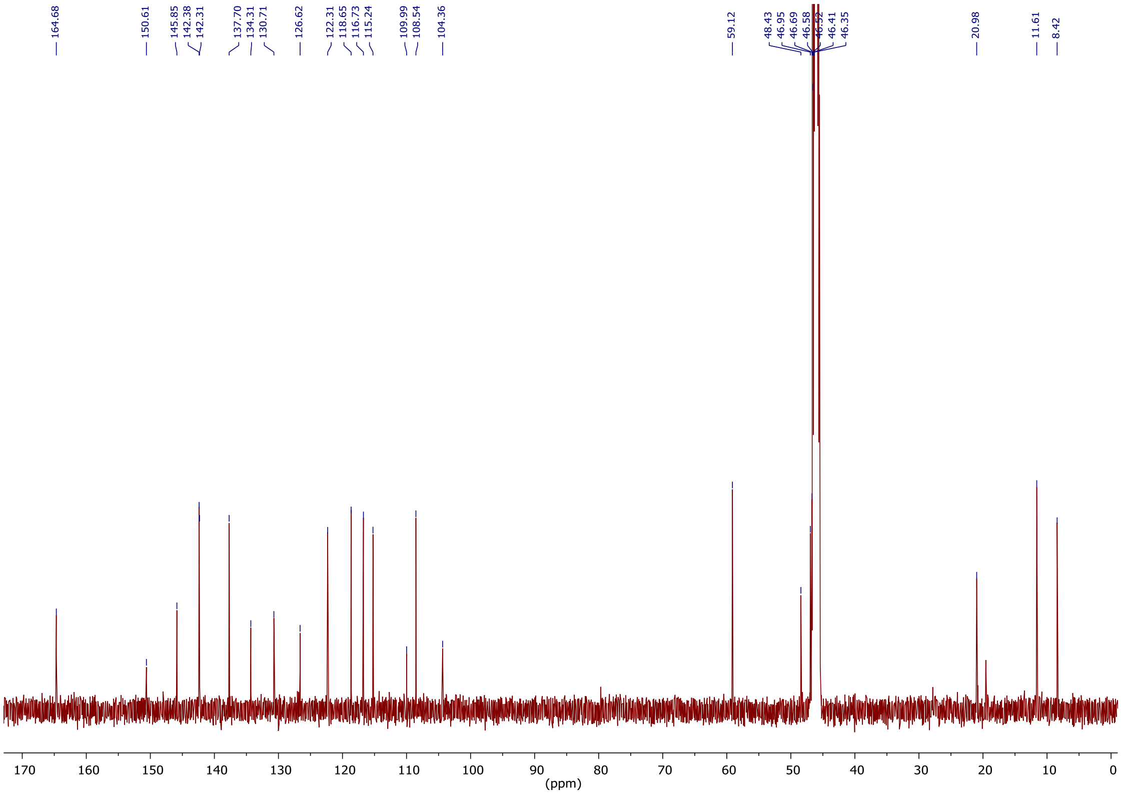

Figure S20. ^{13}C NMR spectrum of compound **11** (MeOD, 126 MHz).

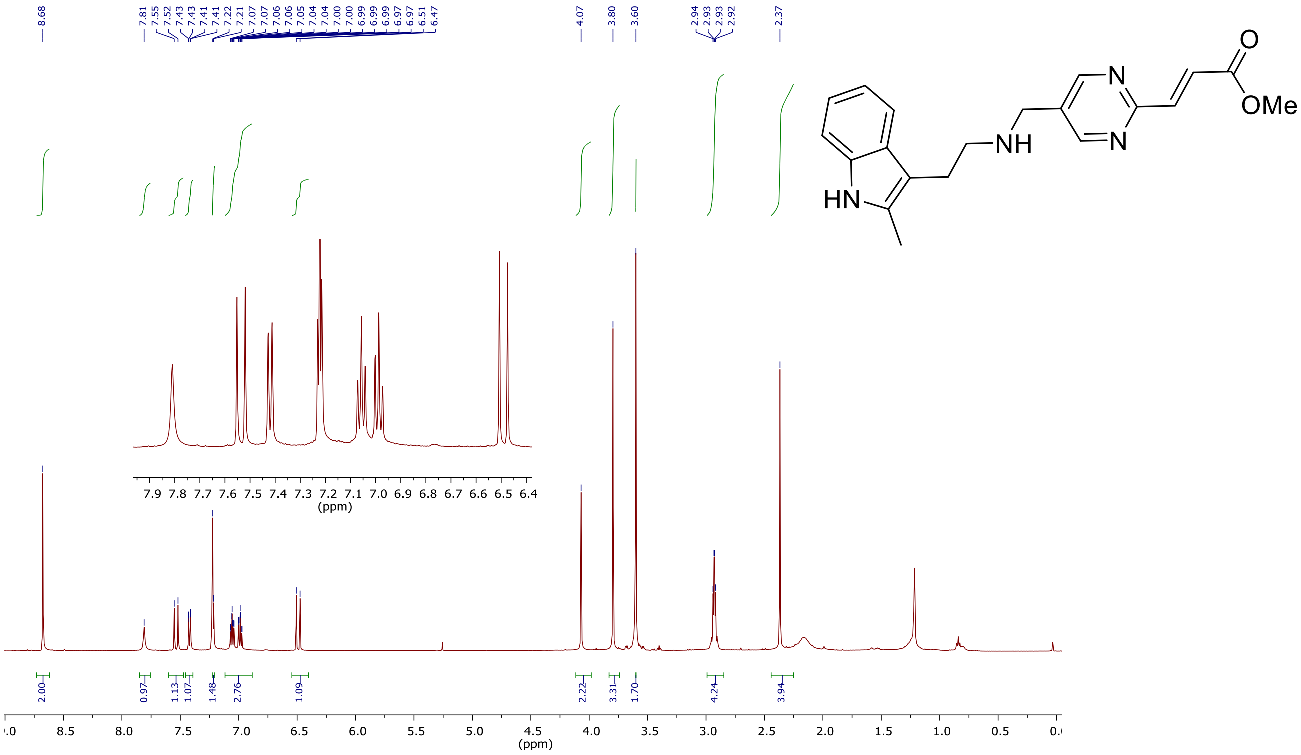

Figure S21. ^{1}H NMR spectrum of compound **22** ($CDCl_3$, 500 MHz).

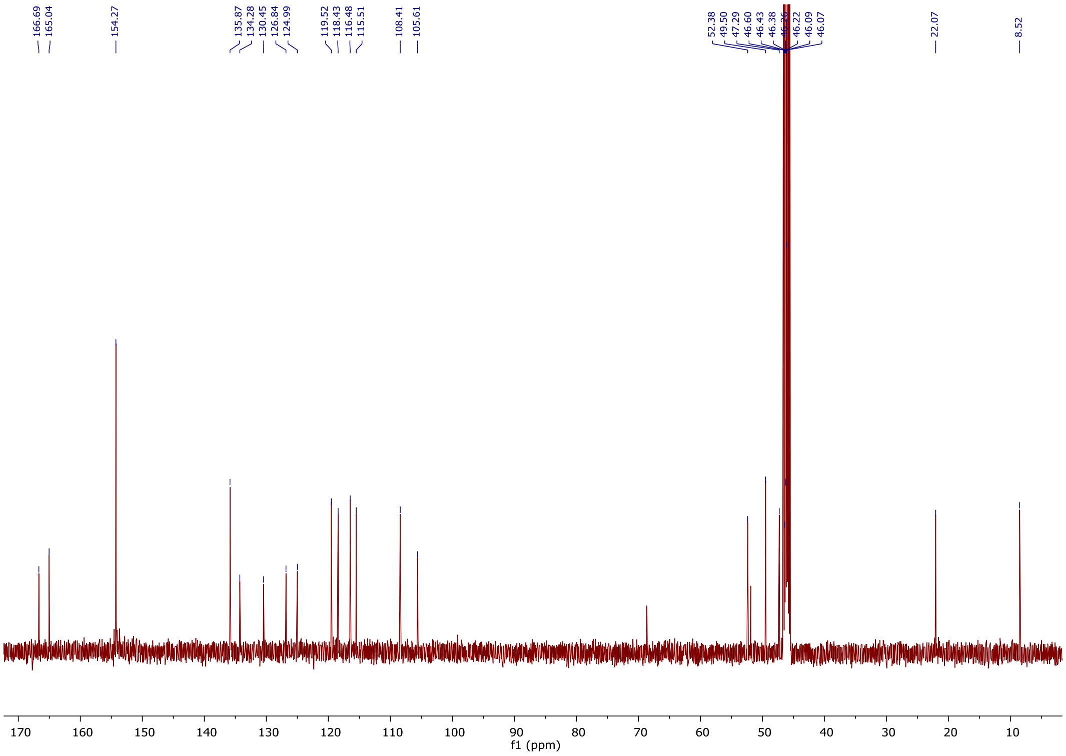

Figure S22. ^{1}H NMR spectrum of compound **22** (MeOD, 500 MHz).

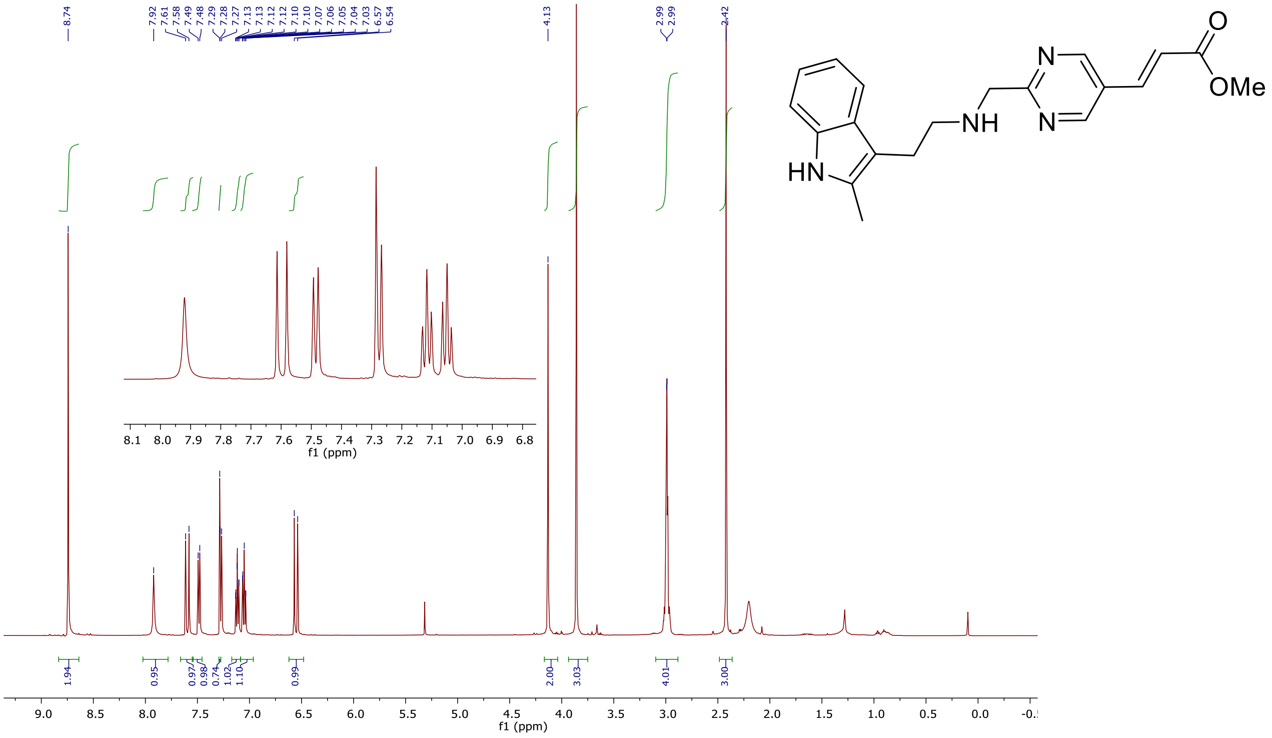

Figure S23. ^{1}H NMR spectrum of compound **23** ($CDCl_3$, 500 MHz).

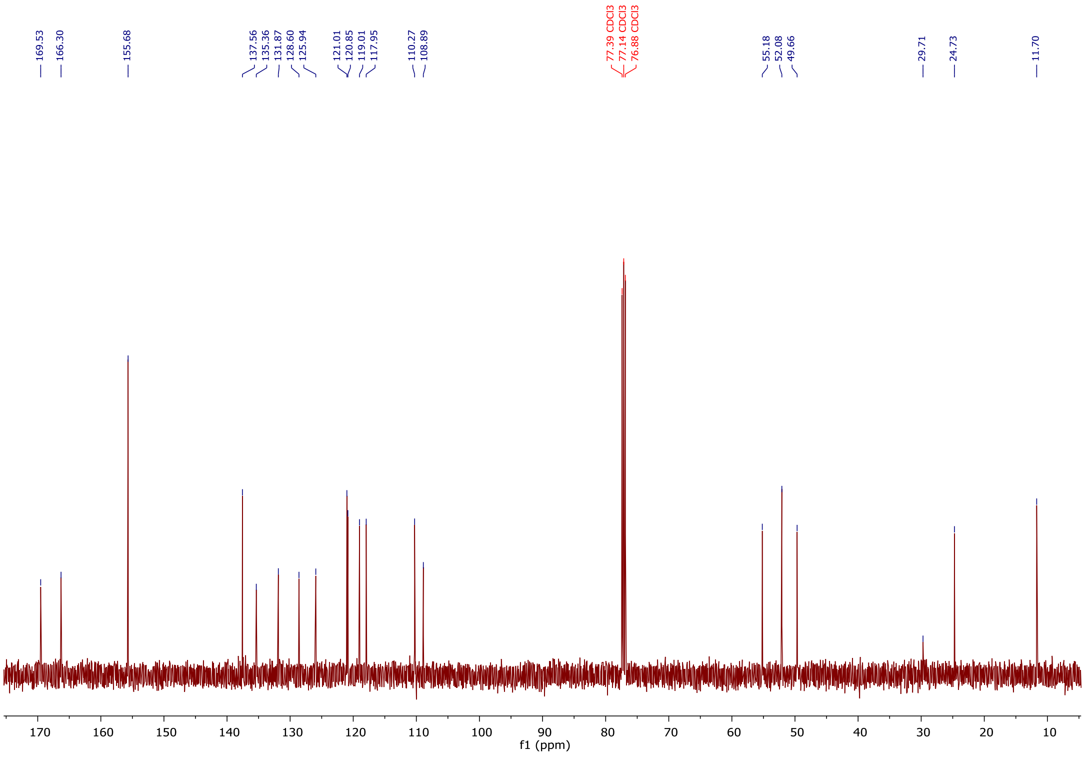

Figure S24. ^{13}C NMR spectrum of compound **23** ($CDCl_3$, 500 MHz).

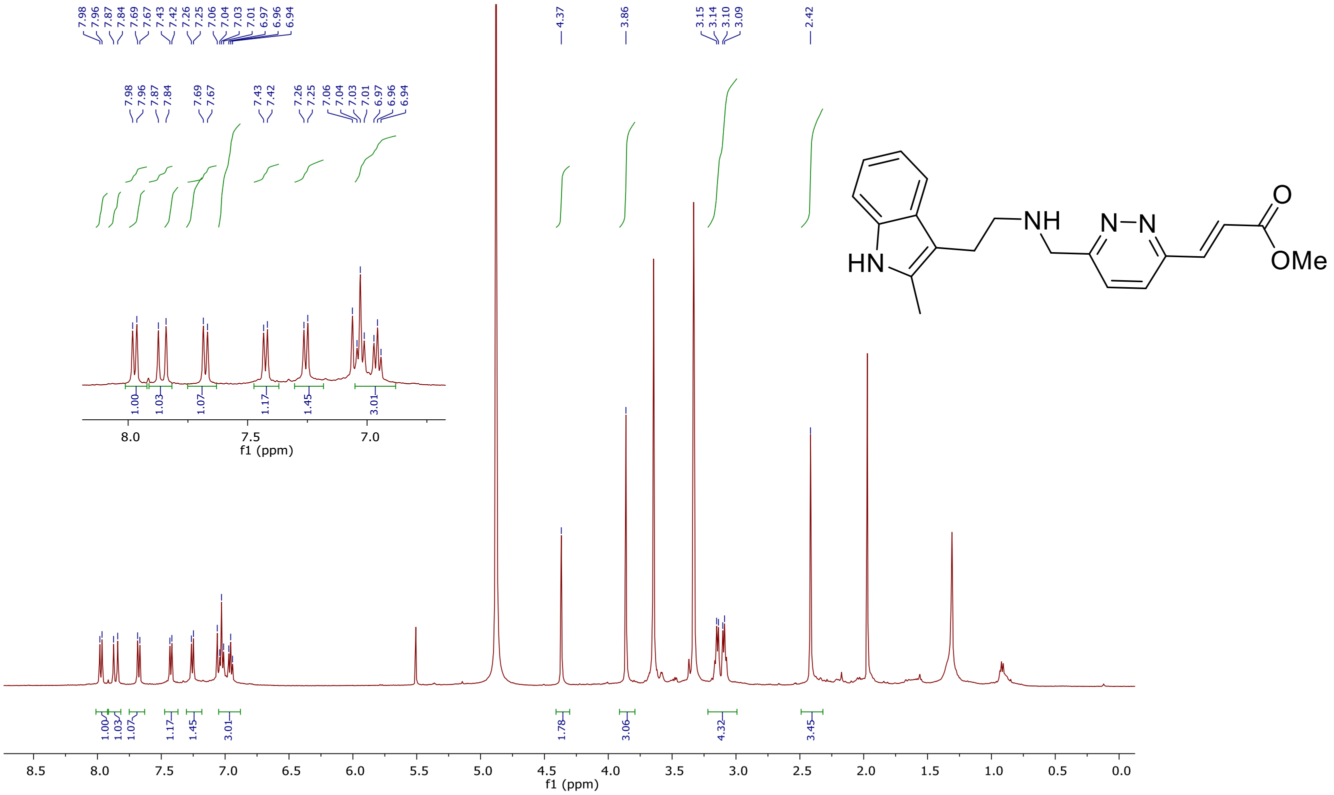

Figure S25. ^{1}H NMR spectrum of compound **26** ($CDCl_3$ + MeOD, 500 MHz).

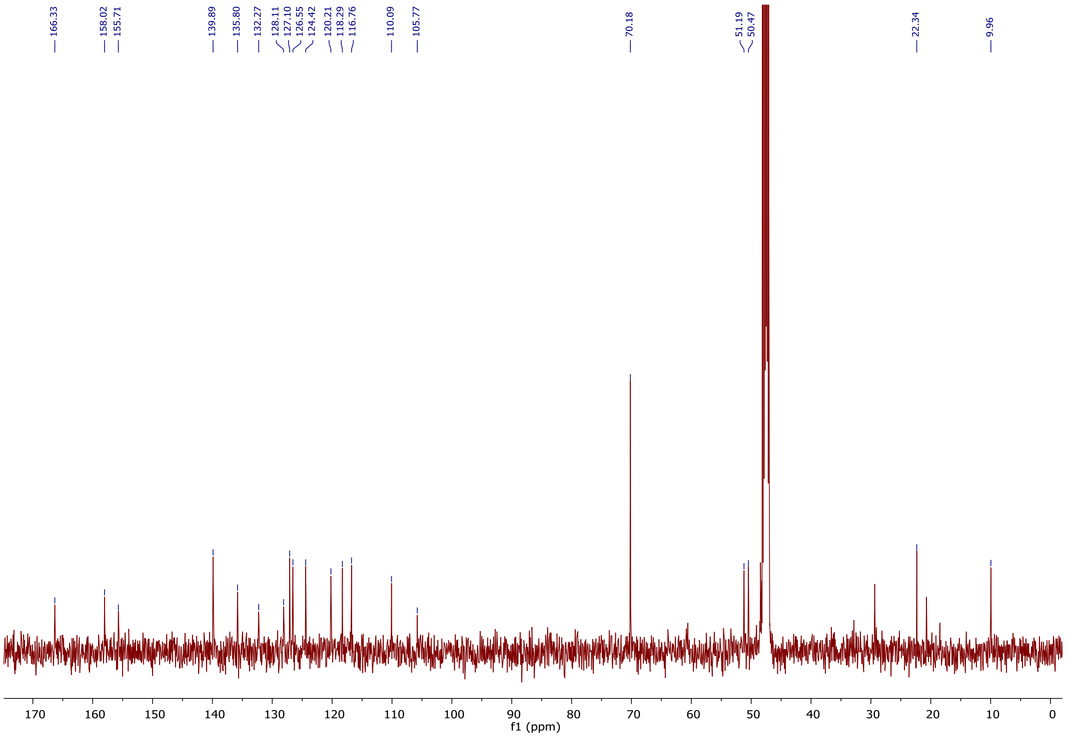

Figure S26. ^{13}C NMR spectrum of compound **26** (MeOD, 126 MHz).

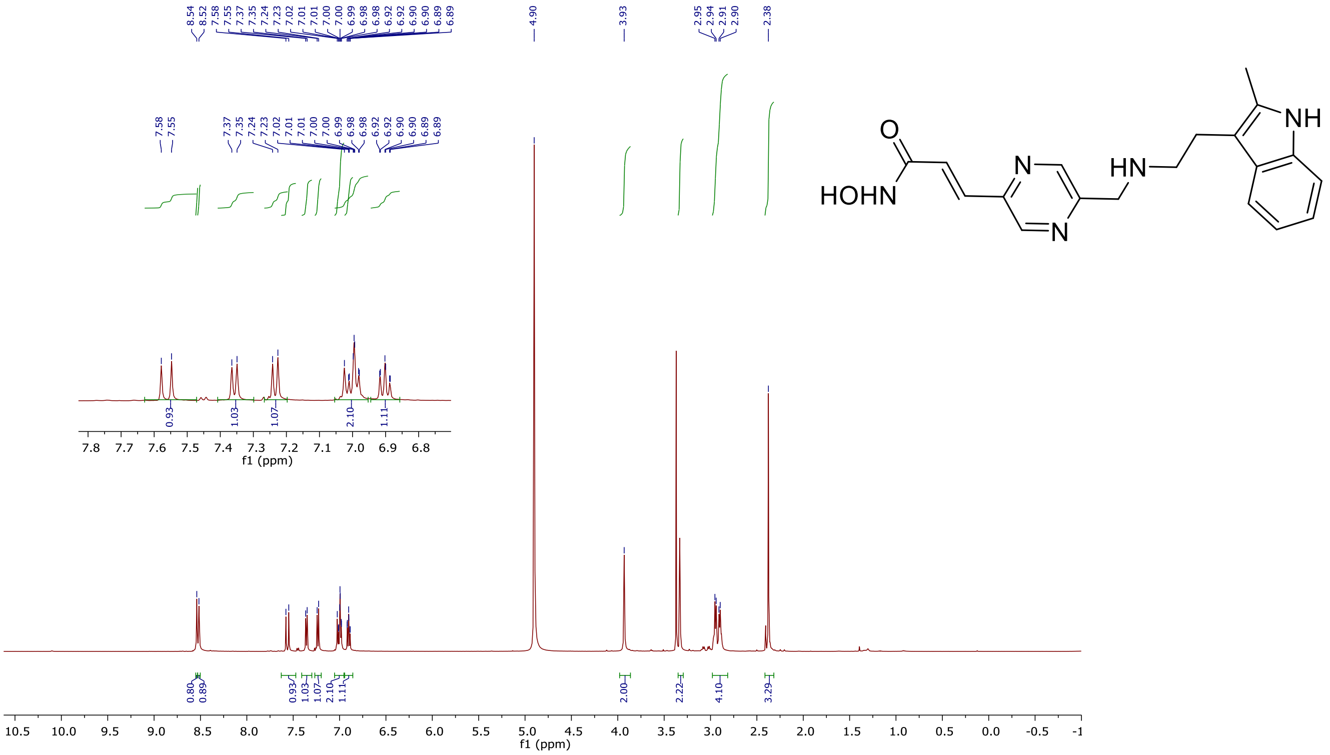

Figure S27. ^{1}H NMR spectrum of compound **TOI1** (MeOD, 500 MHz).

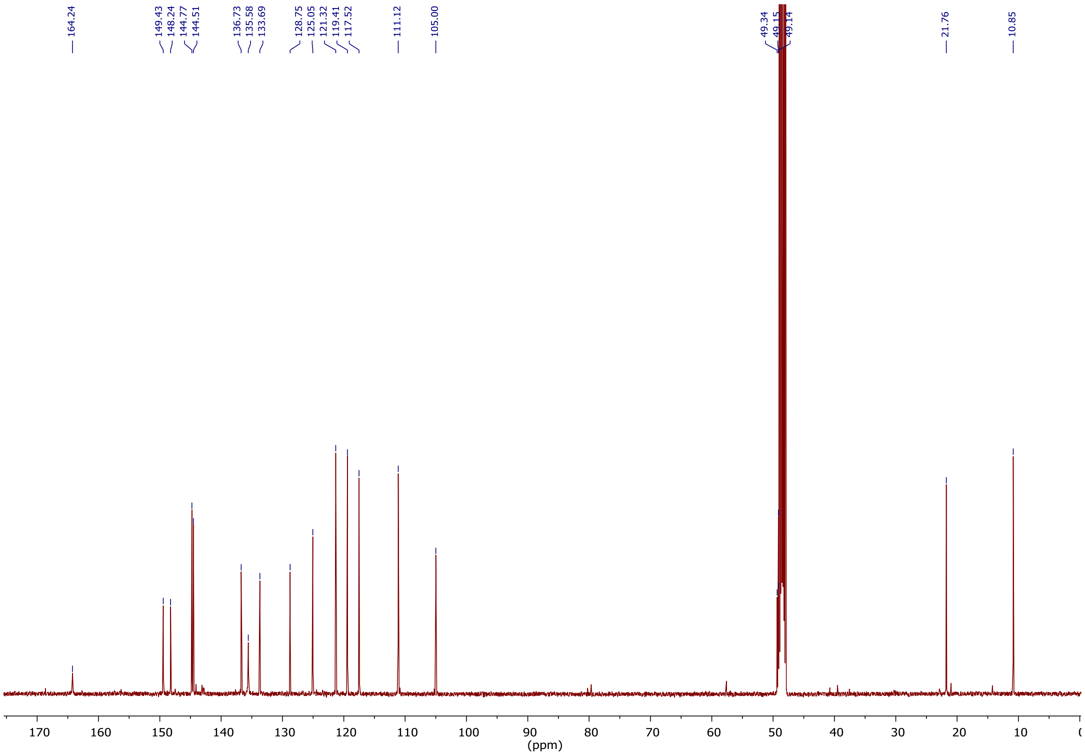

Figure S28. ^{13}C NMR spectrum of compound **TOI1** (MeOD, 126 MHz).

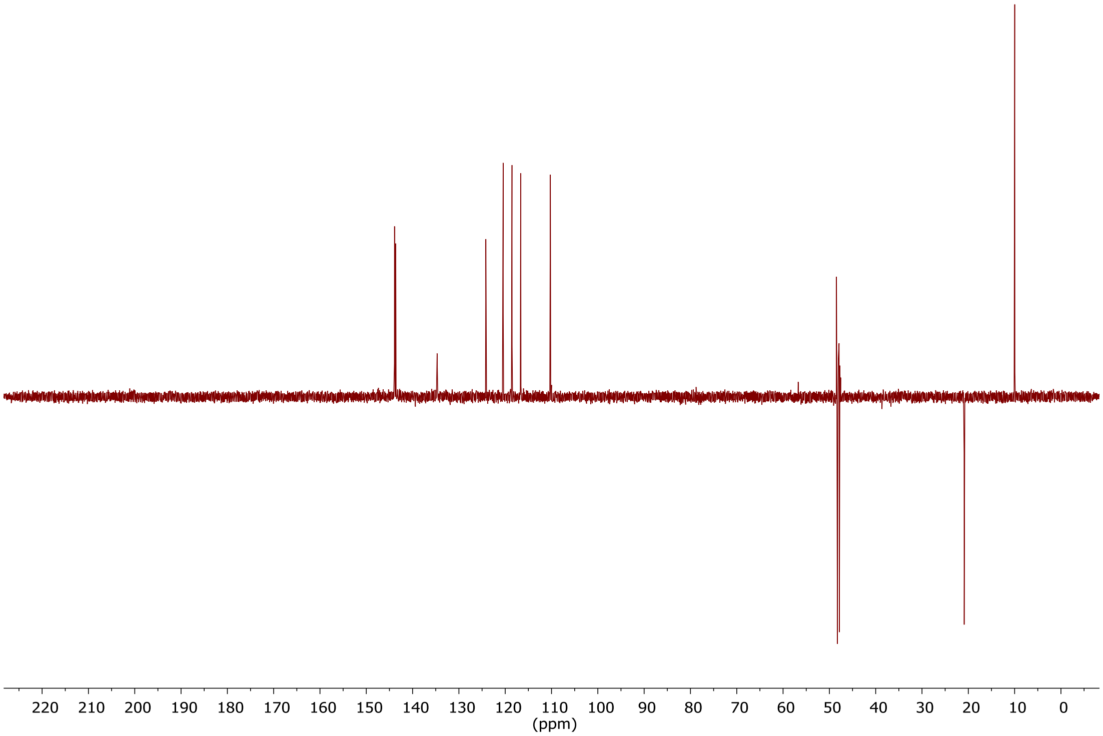

Figure S29. DEPT NMR spectrum of compound **TOI1**.

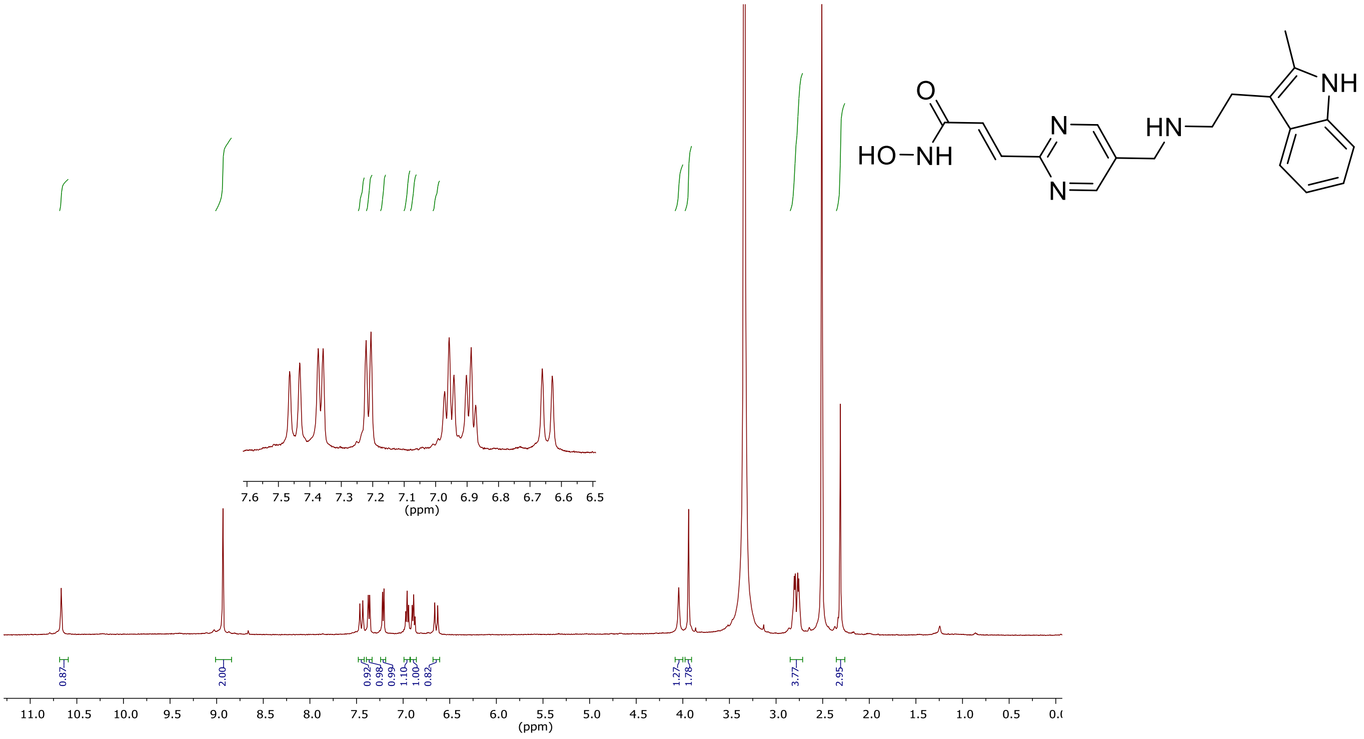

Figure S30. ^{1}H NMR spectrum of compound **TOI2** (DMSO, 500 MHz).

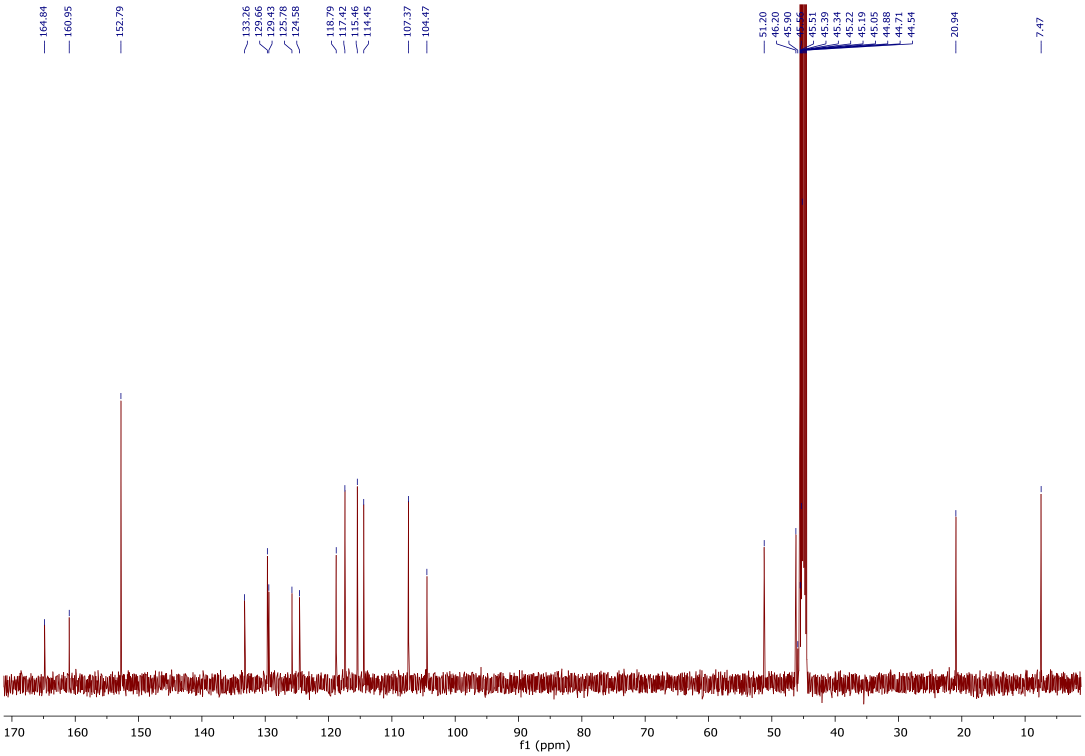

Figure S31. ^{13}C NMR spectrum of compound **TOI2** (MeOD, 126 MHz).

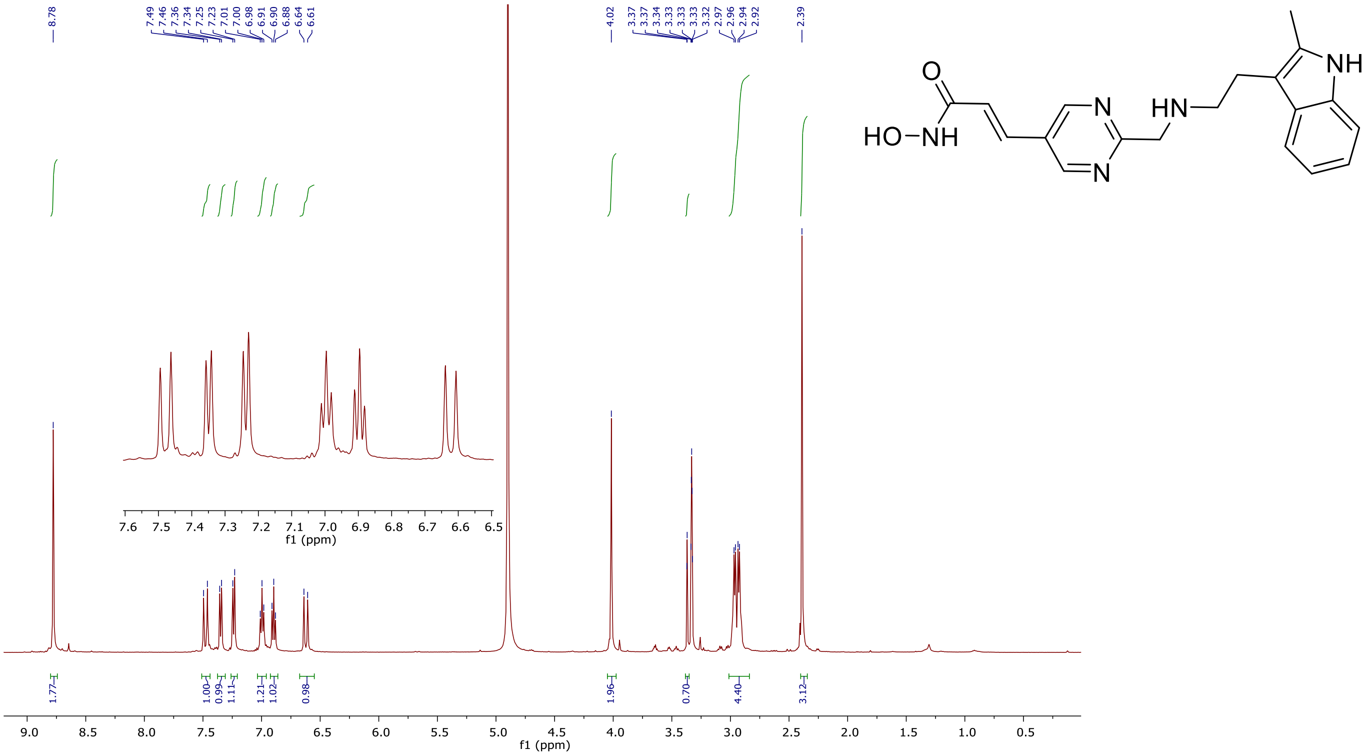

Figure S32. ^{1}H NMR spectrum of compound **TOI3** (MeOD, 500 MHz).

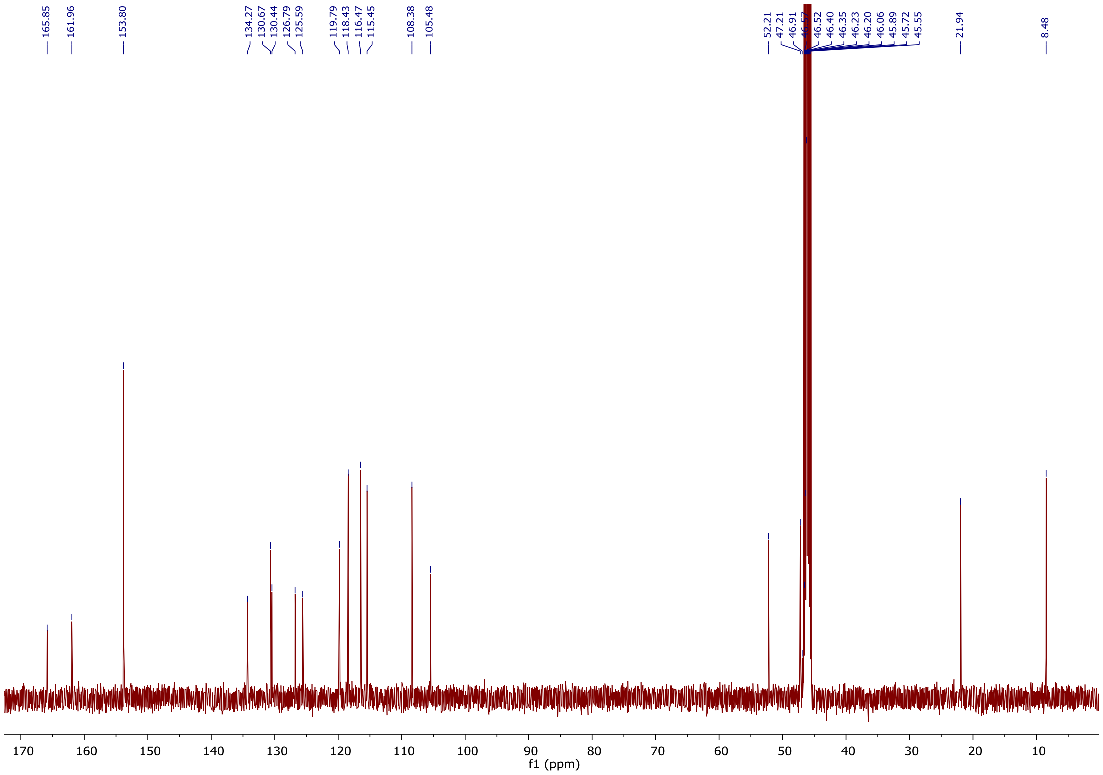

Figure S33. ^{13}C NMR spectrum of compound **TOI3** (MeOD, 126 MHz).

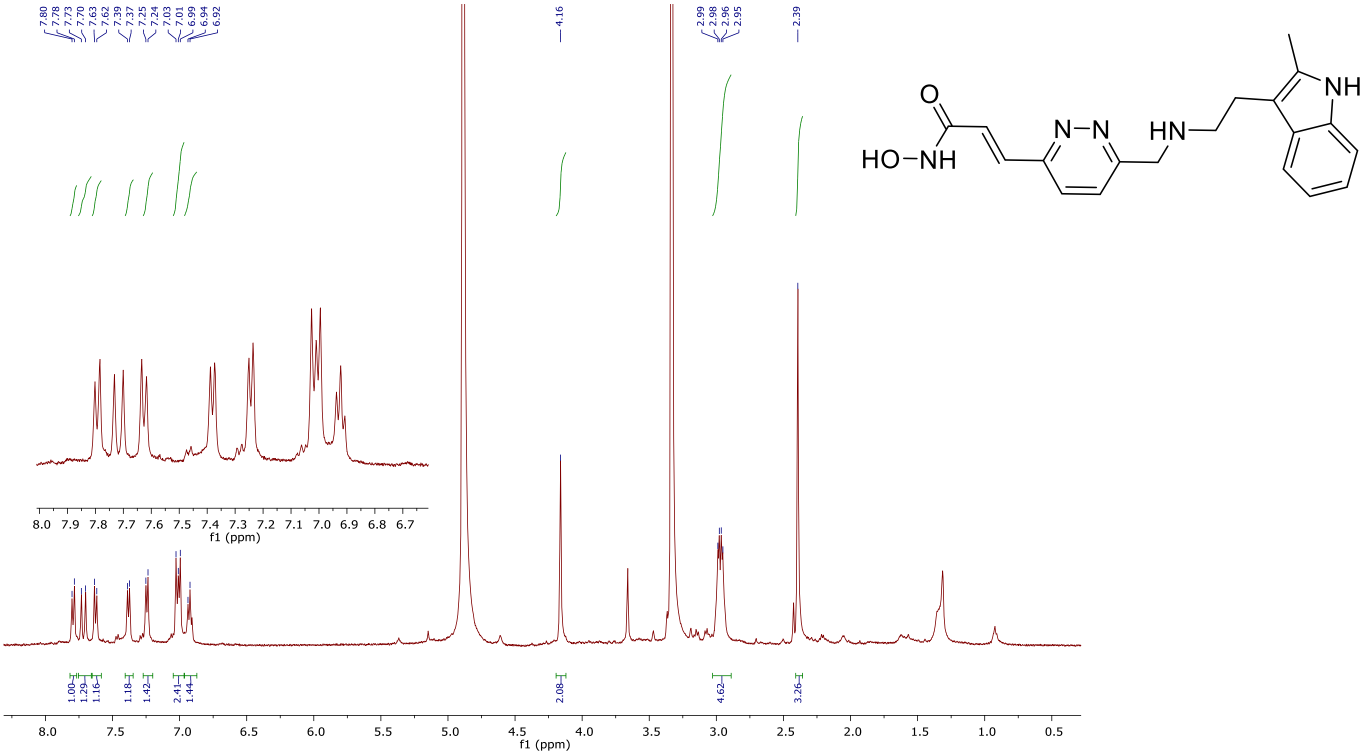

Figure S34. ^{1}H NMR spectrum of compound **TOI4** (MeOD, 500 MHz).

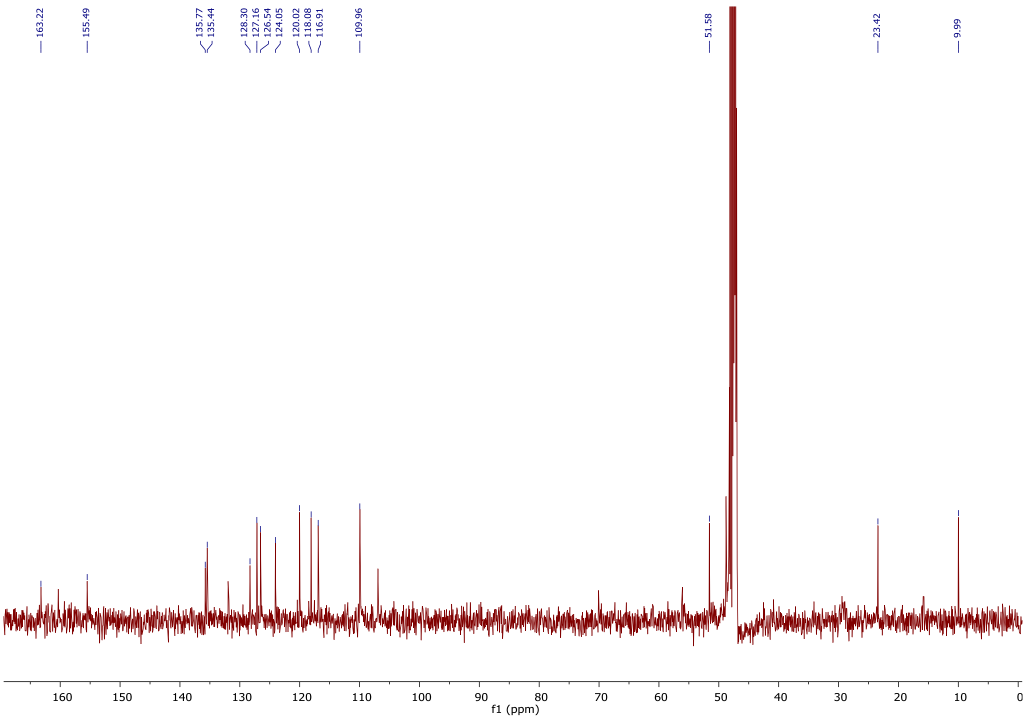

Figure S35. ^{13}C NMR spectrum of compound **TOI4** (MeOD, 126 MHz).

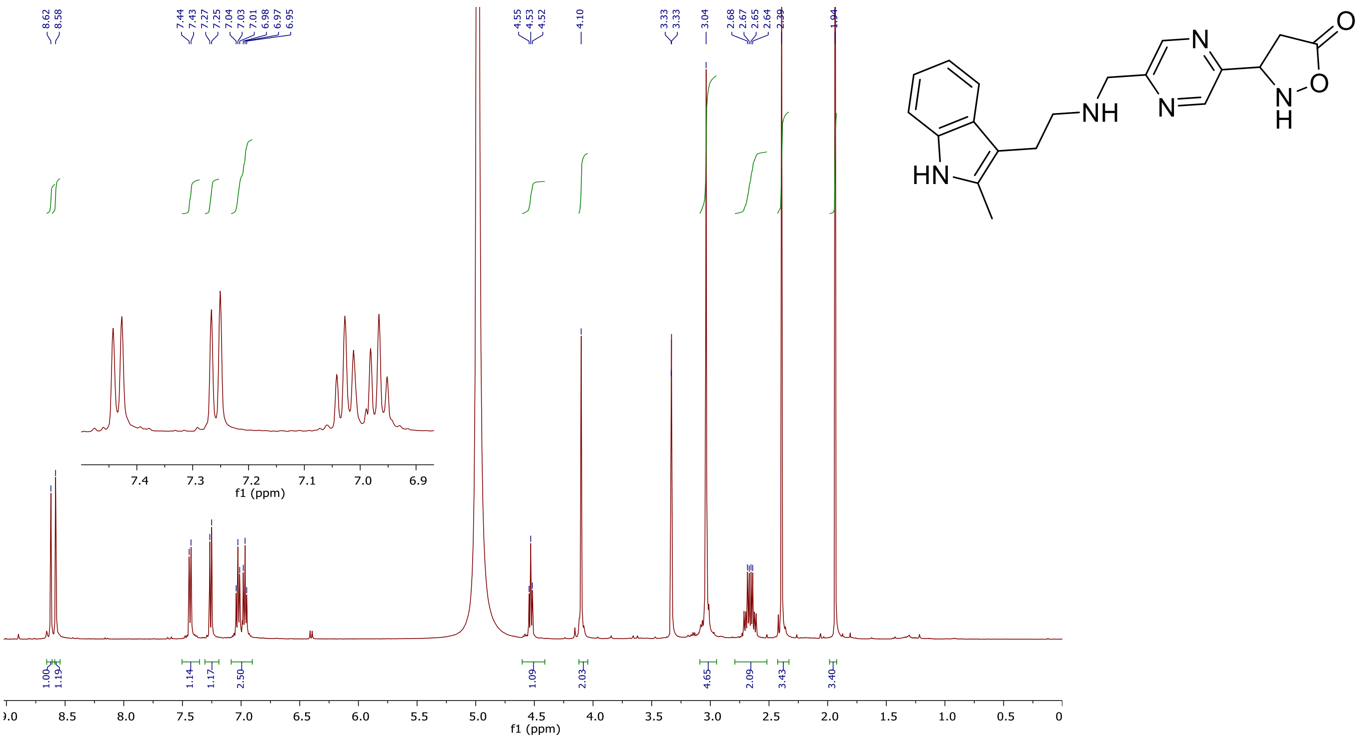

Figure S36. ^{1}H NMR spectrum of compound **12** (MeOD, 500 MHz).

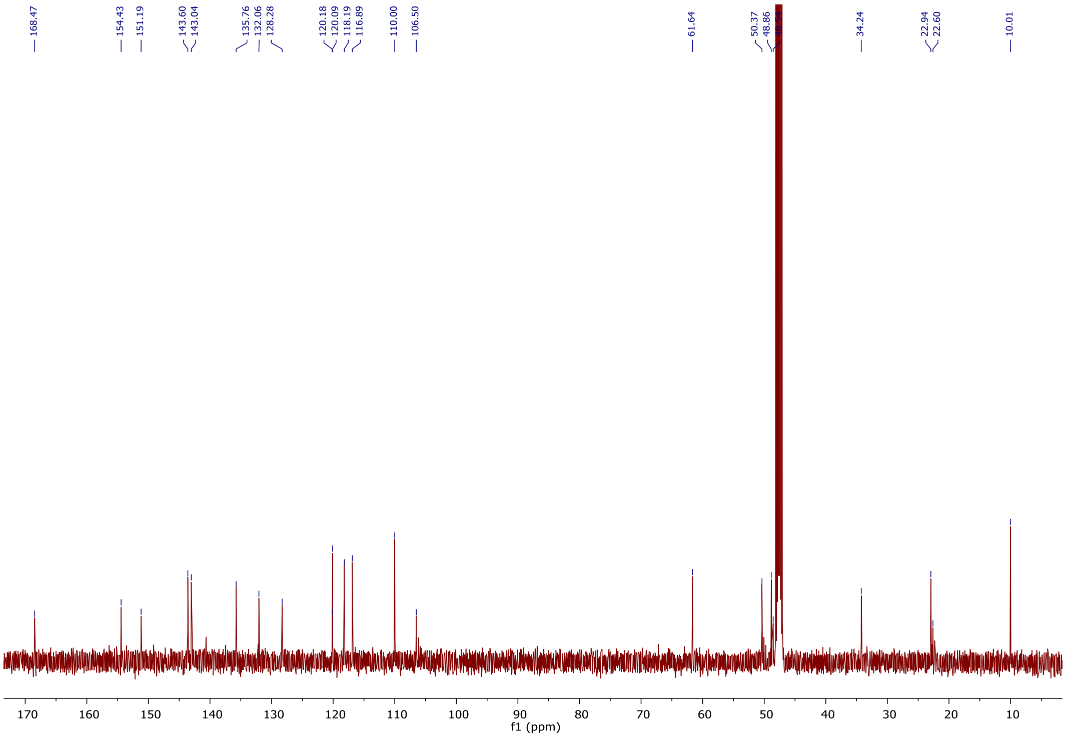

Figure S37. ^{13}C NMR spectrum of compound **12** (MeOD, 125 MHz).

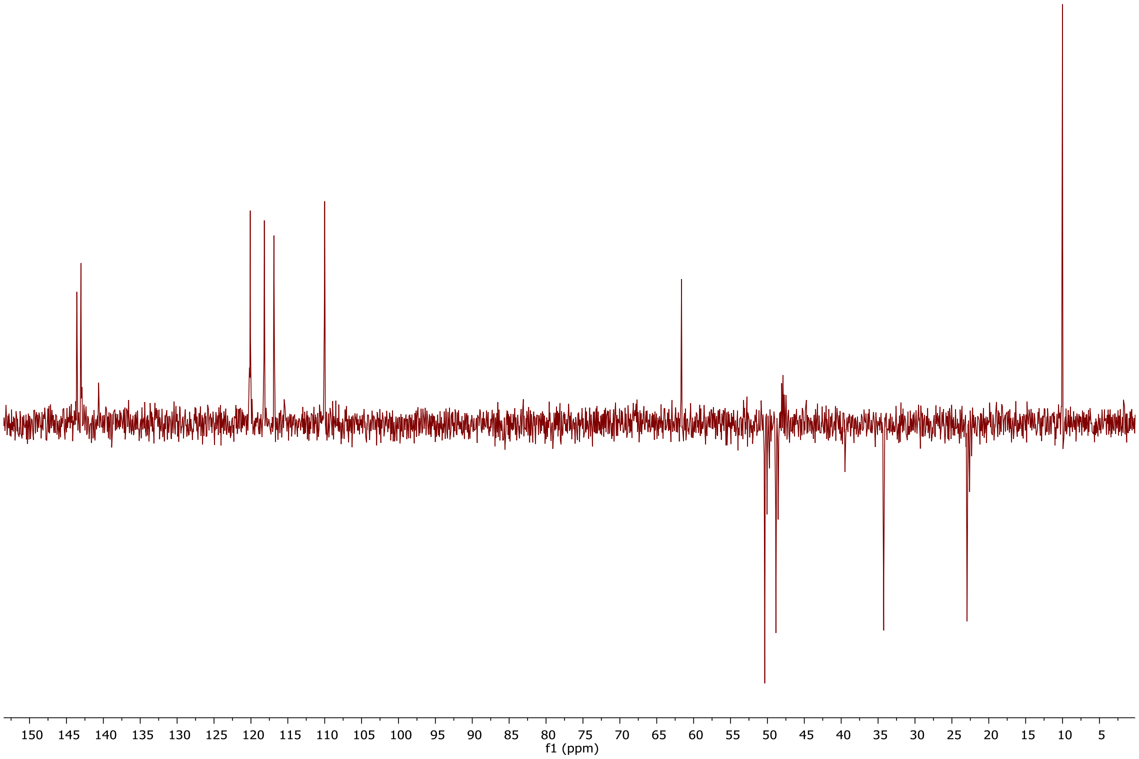

Figure S38. DEPT NMR spectrum of compound **12** in MeOD.

5. References

1. Mamane, V.; Aubert, E.; Fort, Y. *J. Org. Chem.* **2007**, 72, 19, 7294-7300.

2. Zou, Y.; Yan, C.; Zhang, H.; Xu, J.; Zhang, D.; Huang, Z.; Zhang, Y. *Eur. J. Med. Chem*, **2017**, *138*, 313-319
